# Origin and function of activated fibroblast states during zebrafish heart regeneration

**DOI:** 10.1038/s41588-022-01129-5

**Published:** 2022-07-21

**Authors:** Bo Hu, Sara Lelek, Bastiaan Spanjaard, Hadil El-Sammak, Mariana Guedes Simões, Janita Mintcheva, Hananeh Aliee, Ronny Schäfer, Alexander M. Meyer, Fabian Theis, Didier Y. R. Stainier, Daniela Panáková, Jan Philipp Junker

**Affiliations:** 1 Max Delbrück Center for Molecular Medicine in the Helmholtz Association, Berlin Institute for Medical Systems Biology, Berlin, Germany; 2 Max Delbrück Center for Molecular Medicine in the Helmholtz Association, Berlin, Germany; 3 DZHK (German Centre for Cardiovascular Research) partner site, Berlin, Germany; 4 Department of Developmental Genetics, Max Planck Institute for Heart and Lung Research, Bad Nauheim, Germany; 5 DZHK (German Centre for Cardiovascular Research) partner site Rhine/Main, Frankfurt, Germany; 6 Helmholtz Center Munich – German Research Center for Environmental Health, Institute of Computational Biology, Neuherberg, Munich Germany

**Keywords:** Systems analysis, Transcriptomics, Cardiovascular diseases

## Abstract

The adult zebrafish heart has a high capacity for regeneration following injury. However, the composition of the regenerative niche has remained largely elusive. Here, we dissected the diversity of activated cell states in the regenerating zebrafish heart based on single-cell transcriptomics and spatiotemporal analysis. We observed the emergence of several transient cell states with fibroblast characteristics following injury, and we outlined the proregenerative function of collagen-12-expressing fibroblasts. To understand the cascade of events leading to heart regeneration, we determined the origin of these cell states by high-throughput lineage tracing. We found that activated fibroblasts were derived from two separate sources: the epicardium and the endocardium. Mechanistically, we determined Wnt signalling as a regulator of the endocardial fibroblast response. In summary, our work identifies specialized activated fibroblast cell states that contribute to heart regeneration, thereby opening up possible approaches to modulating the regenerative capacity of the vertebrate heart.

## Abstract (web summary)

Single-cell RNA sequencing and spatiotemporal analysis of the regenerating zebrafish heart identify transient proregenerative fibroblast-like cells that are derived from the epicardium and the endocardium. Wnt signalling regulates the endocardial fibroblast response.

## Main

Heart injury in adult mammals typically leads to permanent scarring. However, the adult zebrafish heart regenerates efficiently after injury, making zebrafish the preeminent model system for study of the cellular and molecular mechanisms of heart regeneration^1,2^. After cryoinjury, which mimics aspects of myocardial infarction, the injured zebrafish heart undergoes a transient period of fibrosis^3^, during which the damaged heart muscle regenerates via dedifferentiation and proliferation of cardiomyocytes^4,5^. This process is accompanied by hypoxia-induced revascularization of the newly formed heart muscle tissue^6^. Many pathways and factors involved in heart regeneration have been described^7–11^. Important molecular signals for zebrafish heart regeneration emanate from the epicardium and endocardium^3,11–14^. Other cell types are required for regeneration: inflammation is an early response to cryoinjury, and depletion of macrophages leads to delayed regeneration^15,16^. Moreover, fibroblasts (potentially of different origins) are activated after injury, and ablation of fibroblasts leads to reduced cardiomyocyte proliferation^17^. Altogether, these data suggest that cell types or transient activated cell states residing in the regenerative niche may be important cellular regulators of regeneration. A number of recent single-cell RNA sequencing (scRNA-seq) studies have determined cell type diversity in the mammalian heart^18–21^, as well as in the regenerating zebrafish heart^22,23^. However, to our knowledge, there has been no systematic data-driven attempt to identify proregenerative cell states and their cell type of origin. Consequently, our knowledge of the cellular composition of the regenerative niche and the underlying signalling interactions remains incomplete. Current definitions of activated macrophages and fibroblasts rely heavily on transgenes, may be affected by observation bias and probably underestimate the complexity of cell states involved in regeneration.

## Results

### The cellular composition of the regenerating heart

**Fig. 1:**
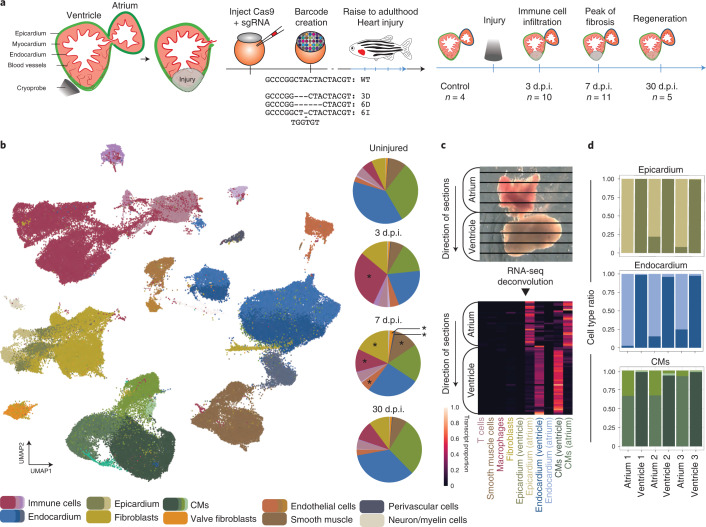
The cellular composition of the regenerating heart. **a**, Cartoon of the experimental approach. Cells were barcoded with indels (insertions or deletions) during early development, and fish were raised to adulthood. Hearts were harvested either as an uninjured control or at 3, 7 or 30 d.p.i. **b**, UMAP representation of single-cell RNA-seq data and clustering results. Pie charts show the proportions of different cell types at different time points after injury. In the pie chart representation, similar cell types are grouped and shown by one (representative) colour. Asterisks denote cell types with a statistically significant change in proportions compared with uninjured controls. **c**, Mapping of single-cell data onto a spatially resolved tomo-seq data set. A computational deconvolution approach revealed chamber-specific cell subtypes. **d**, Distribution of subtypes of cardiomyocytes (CMs), endocardial cells and epicardial cells for scRNA-seq data sets in which atrium and ventricle were physically separated. Colour scheme as in Supplementary Fig. 3. Source data

For systematic identification of cardiac cell types in the healthy and regenerating zebrafish heart, we performed scRNA-seq of around 200,000 dissociated cells at different stages before and after injury (Fig. 1a). To limit experimental biases, we did not apply any sorting procedure. To include information about the developmental origin of cells, we applied a method for massively parallel lineage tracing based on CRISPR–Cas9 technology^24–26^. By injecting Cas9 and a single-guide RNA (sgRNA) against a multicopy transgene (*dTomato* in the zebrabow^27^ line), we recorded lineage relationships in early development by creating ‘genetic scars’ that serve as lineage barcodes^24^ (Methods).

We first assessed cell type diversity in the healthy and regenerating heart. Clustering of single-cell transcriptomes revealed all major cardiac cell types (Fig. 1b, Supplementary Figs. 1 and 2 and Supplementary Data Files 1 and 2). As expected, we observed a strong increase in fibroblasts and immune cells after injury (Fig. 1b). Closer inspection of the clustering data revealed a substructure among the cell types of the three main layers of the heart: epicardium, myocardium and endocardium (Supplementary Fig. 3a–c and Supplementary Data File 2). We hypothesized that this cell type substructure might correspond to spatial differences due to the functional specifications of these cell types in the atrium and ventricle. Using the tomo-seq method for spatially resolved transcriptomics^28^, and deconvolving the spatial data into single-cell transcriptional profiles^29^, we could validate atrial and ventricular enrichment for some of these cell subtypes (Fig. 1c, Supplementary Fig. 3a–c and Methods). We confirmed this finding by physical separation of the atrium and ventricle, followed by scRNA-seq (Fig. 1d).

### Cell type diversity of cardiac fibroblasts

**Fig. 2:**
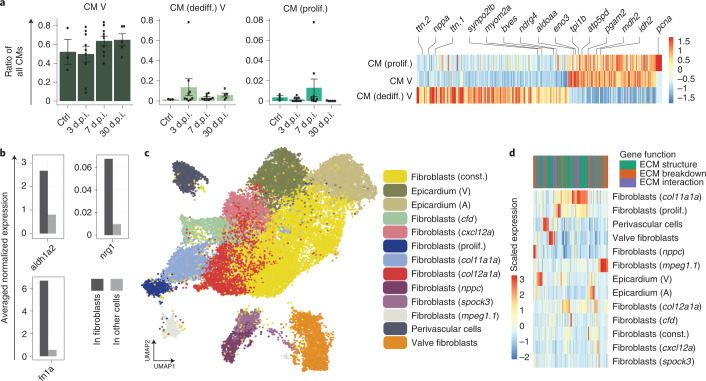
Cell type diversity of cardiac fibroblasts. **a**, Left: relative changes in abundance of different subtypes of CMs across the time points (*n* = 3, 9, 9, 5 animals; error bars show s.e.m.). Right: differentially expressed genes between subtypes of CMs. **b**, Comparison of average normalized expression of known proregenerative factors in fibroblasts and other cell types. The comparison used data pooled over all time points, and fibroblasts were defined as all cells in the yellow cluster in Fig. 1b. **c**, UMAP representation of the subclustering of *col1a1a*-expressing cells. **d**, Expression of ECM-related genes in different fibroblast cell types. The genes were classified according to their contribution to structure, breakdown or interaction of the ECM. A, atrium; Ctrl., control; const., constitutive; dediff., dedifferentiated; prolif., proliferating; V, ventricle. Source data

We identified a further transcriptional substructure among the cardiomyocytes (Fig. 2a). In addition to adult cardiomyocytes, which are characterized by expression of genes involved in ATP synthesis and the tricarboxylic acid cycle (*atp5pd* and *aldoaa*), we detected a smaller cluster characterized by genes associated with cardiomyocyte development (ttn.1, ttn.2, *bves* and *synpo2lb*), as well as *nppa*, which has previously been shown to be a marker for dedifferentiated cardiomyocytes in the border zone^22^. These dedifferentiated cardiomyocytes, which are a hallmark of the regenerating heart, had increased in number by 3 d.p.i. (days postinjury) (Fig. 2a) and partially colocalized with the established marker gene *nppa*^22^ at 7 d.p.i. (Supplementary Fig. 3d).

We noticed that three well-established signalling factors in heart regeneration were strongly enriched in fibroblasts: *aldh1a2* (ref. ^7^), which is the enzyme synthesizing retinoic acid; the cardiomyocyte mitogen *nrg1* (ref. ^30^); and the proregenerative extracellular matrix (ECM) factor *fn1a*^31^ (Fig. 2b). This prompted us to investigate the diversity of cardiac fibroblasts in more detail. Subclustering revealed an unexpectedly large degree of diversity, with 13 transcriptionally distinct clusters of fibroblasts (Fig. 2c, Supplementary Fig. 4a,b and Supplementary Data File 3). These 13 clusters exhibited pronounced differences in their expression profiles of ECM-related genes (Fig. 2d), but their transcriptomic diversity extended far beyond that – the same fibroblast subtypes could be identified with high accuracy after removal of ECM-related genes (Supplementary Fig. 5).

### Identification of proregenerative cardiac fibroblasts

**Fig. 3:**
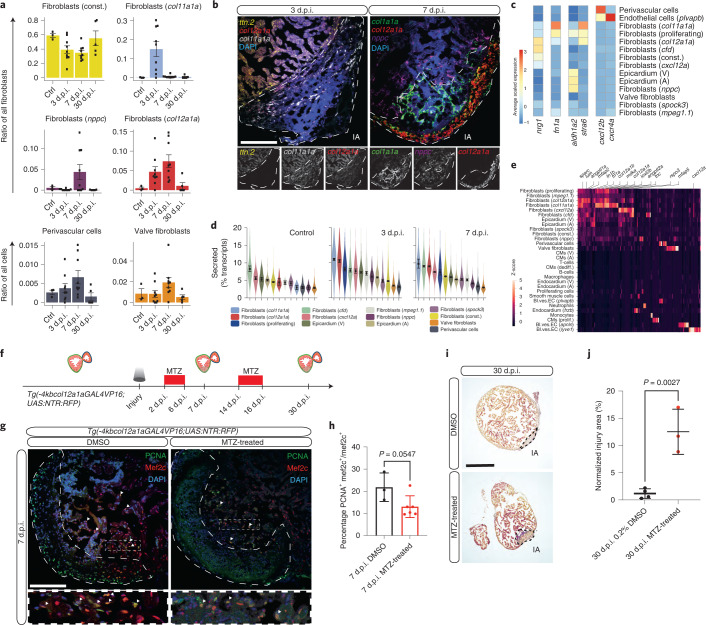
Identification of proregenerative cardiac fibroblasts. **a**, Cell number dynamics of selected fibroblast subclusters across the time points (*n* = 3, 9, 9, 5 animals; error bars show s.e.m.). **b**, Fluorescence in situ hybridization of marker genes. Left panel: const. fibroblasts (green), *col12a1a* fibroblasts (red) and *nppc* fibroblasts (purple) at 7 d.p.i. Right panel: *col12a1a* fibroblasts (red), *col11a1a* fibroblasts (white) and dedifferentiated CMs expressing *ttn.2* (yellow) at 3 d.p.i. Injury areas (IA) are indicated with a dashed white line. Scale bar, 100 μm. **c**, Average expression of selected signalling genes in fibroblast subclusters. Blood vessel endothelial cells are included to show their interaction with perivascular cells via Cxcl12b–Cxcr4a signalling. **d**, Mean secretome expression of fibroblast cell types (*n* = 1,947, 8,611 and 17,261 cells; error bars indicate 3 × s.e.m.; *y* axis truncated for readability; full plots in Supplementary Fig. 10a). **e**, Differentially expressed secretome genes at 3 d.p.i. Genes with a reported function in regeneration, morphogenesis, tissue development or angiogenesis are highlighted (Bl. ves. EC: Blood vessel endothelial cells). **f**, Schematic of the ablation experiment for *col12a1a*-expressing cells. **g**, Immunostaining of sections of cryoinjured hearts of *Tg(-4kbcol12a1aGAL4VP16;UAS:NTR:RFP)* zebrafish treated with DMSO and MTZ at 7 d.p.i.; sections stained for Mef2c (CMs, red), PCNA (proliferation marker, green) and DNA (DAPI, blue). Arrowheads point to PCNA^+^ CMs; white dashed lines indicate IA. Scale bar, 100 μm. **h**, Percentages of PCNA^+^ CMs in DMSO-treated fish (*n* = 3) and MTZ-treated fish (*n* = 6) at 7 d.p.i. *n* represents biologically independent samples from two independent experiments. Data are shown as mean and s.d. Two-tailed unpaired Student’s *t* test, *P* = 0.0547. **i**, Histological comparison of the IA at 30 d.p.i. with and without MTZ treatment. Scale bar, 300 μm. **j**, Relative size of the IA across all histological replicates at 30 d.p.i. in 0.2% DMSO- (*n* = 4) and MTZ-treated (*n* = 3) samples. *n* represents biologically independent samples from two independent experiments. Data are shown as mean and s.d. Two-tailed unpaired Student’s *t* test, *P* = 0.0027. Source data

To focus our analysis on those fibroblast subtypes that may be part of the regenerative niche, we analysed the dynamics of the cell clusters after injury. Three clusters of fibroblasts, characterized by expression of *col11a1a*, *col12a1a* and *nppc*, respectively, were transiently present at the peak of regeneration (3 and 7 d.p.i.) but virtually absent before injury and after regeneration (Fig. 3a, Supplementary Fig. 4c and Supplementary Data File 4). Owing to their transient nature, we refer to these three clusters as cell states instead of cell types. Notably, *col12a1a*, a nonfibrillar collagen that may act as a matrix-bridging component, has already been shown to be expressed in epicardial and connective tissues following heart injury^32^ and is known to be involved in regeneration of other organ systems in zebrafish^33^. Here, *col12a1a* was not only expressed in *col12a1a* fibroblasts but also in *col11a1a* fibroblasts, and these two transient fibroblast subtypes exhibited similar expression profiles overall (Supplementary Fig. 6). Two other cell types with ECM-related functions, perivascular cells and valve fibroblasts, also showed increased abundance after injury (Fig. 3a, Supplementary Fig. 4c and Supplementary Data File 4). Other fibroblast types showed only moderate changes after injury. Importantly, established markers for ‘activated’ fibroblasts including *postnb*^17^ captured some but not all fibroblast clusters that are generated following injury and were also expressed in nonfibroblast populations including epicardial cells (Supplementary Fig. 6), suggesting that previous marker-based analyses have underestimated the transcriptional diversity of the cardiac fibroblast population.

To spatially resolve the identified fibroblasts, we performed fluorescence in situ hybridization (Fig. 3b and Supplementary Figs. 7 and 8). This analysis confirmed the location of the transient fibroblast cell states in the border zone as well as the injury area. We next wanted to understand which of the transient fibroblasts expressed signalling factors involved in heart regeneration (Fig. 3c and Supplementary Fig. 9). We found particularly high expression of *nrg1* in *col12a1a* fibroblasts, whereas *fn1a* was expressed almost exclusively in *col11a1a* fibroblasts. Furthermore, we observed that retinoic acid *(aldh1a2)* was produced at high levels by *nppc* fibroblasts as well as in the epicardium (Supplementary Fig. 8) and at lower levels in the endocardium (Supplementary Fig. 9). The retinoic acid readout gene *stra6 (stimulated by retinoic acid 6)*^34,35^ was expressed highly in *col11a1a*, *col12a1a* and proliferating fibroblasts (but not in cardiomyocytes), suggesting possible communication between these cell types. Moreover, we noticed a very specific interaction between perivascular cells and blood vessel endothelium via Cxcl12b–Cxcr4a chemokine signalling. Perivascular cells are known regulators of blood vessel formation^36^, and it was recently shown that Cxcl12b–Cxcr4a signalling is important for revascularization of the regenerating heart^6^.

We reasoned that a potential proregenerative function of transient cell states might be driven by secreted factors. Bioinformatic analysis revealed that expression of secretome genes increased in regenerating hearts at 3 d.p.i. and 7 d.p.i. compared with uninjured control hearts (Fig. 3d and Supplementary Fig. 10a). The *col11a1a* and *col12a1a* fibroblasts had the highest secretome expression of all detected cell clusters, and their secretome was enriched in genes with known functions in regeneration, morphogenesis and tissue development (Fig. 3e, Supplementary Fig. 10b and Supplementary Data File 5). The secretome at 3 d.p.i. was also enriched in genes with functions in angiogenesis (for example, *angptl1a* and *angptl2a*), suggesting a possible role of these cell types in revascularization. We next performed ligand–receptor analysis of the identified cell clusters (Methods). We found that the number of putative cell–cell interactions increased drastically after injury and peaked at 3 d.p.i., with a noticeable enrichment of both incoming (receptor) and outgoing (ligand) cell interactions in fibroblast subtypes (Supplementary Fig. 11).

Although our spatiotemporal analysis of single-cell gene expression profiles strongly suggested a proregenerative function of transient fibroblasts, additional experiments were needed to validate this hypothesis. To functionally assess the role of the transient *col11a1a* and *col12a1a* fibroblasts, we applied targeted genetic cell ablation using the nitroreductase/metronidazole (NTR/MTZ) system^37^. We generated and characterized a transgenic line labelling *col12a1a*-expressing fibroblasts, *Tg(-4kbcol12a1a:GAL4VP16;UAS:NTR:RFP)*, subsequently referred to as *col12a1a*>NTR:RFP (Supplementary Fig. 12a–d and Methods). At 7 d.p.i., MTZ-treated *col12a1a*>NTR:RFP fish displayed a marked reduction in RFP expression, in line with our experimental design (Supplementary Fig. 12e). Whereas the injury area in *col12a1a*-depleted hearts at 7 d.p.i. looked normal overall (Supplementary Fig. 12f), five of six hearts displayed reductions in cardiomyocyte proliferation upon depletion of *col12a1a*-expressing cells compared with the vehicle control (Fig. 3g,h). At 30 d.p.i., we found that heart regeneration was significantly impaired by *col12a1a*^*+*^ cell ablation (Fig. 3i,j), suggesting that *col12a1a*-expressing fibroblasts contribute to regeneration. Although it was not possible to formally rule out the possibility that depletion of *col12a1a*-expressing cells may also lead to some tissue degradation of the ventricle independent of a regeneration defect, such effects would be expected to be minor, owing to the lack of *col12a1a* reporter expression in the uninjured ventricle (Supplementary Fig. 12c) and the lack of any visible degeneration outside the injury site (Supplementary Fig. 12e).

In summary, we identified three fibroblast states with potential proregenerative roles during heart regeneration: *col11a1a*, *col12a1a* and *nppc* fibroblasts. Our genetic cell ablation data strongly indicate a role for *col12a1a*-expressing cells in the regenerative niche. However, from which cell types these activated cell states are derived remains to be determined.

### Identification of epicardial fibroblasts

**Fig. 4:**
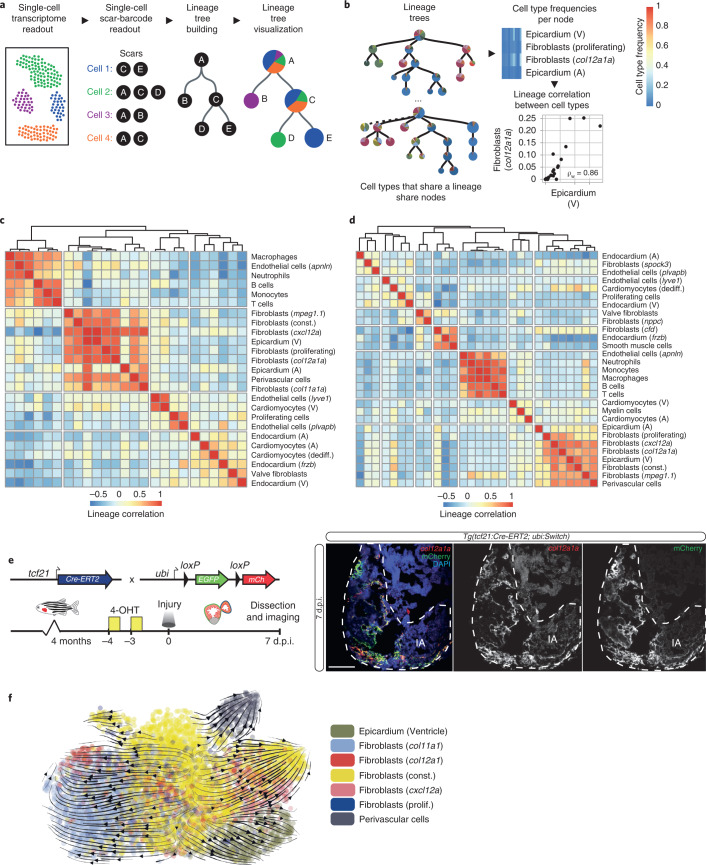
Identification of epicardial fibroblasts. **a**, Cartoon of lineage tree construction using LINNAEUS. **b**, Weighted correlations of cell types over the tree were calculated to quantify lineage similarity. **c**,**d**, Clustering by lineage correlations at 3 d.p.i. (**c**) and 7 d.p.i. (**d**) revealed the epicardial origin of many niche fibroblasts. **e**, Cre–*lox* lineage tracing confirmed the epicardial origin of *col12a1a*-expressing cells. Scale bar, 100 μm. **f**, Trajectory analysis suggested constitutive fibroblasts as the source of *col11a1a* and *col12a1a* fibroblasts at 3 d.p.i. Source data

We next aimed to elucidate the origins of the transient fibroblast states in order to better understand their mechanism of activation. Trajectory inference using partition-based graph abstraction (PAGA)^38^ suggested a dense network of possible transitions between all fibroblast subtypes (Supplementary Fig. 13). We reasoned that the large number of putative trajectories might be a computational artifact caused by a multitude of transcriptionally similar cell types, which might be generated by convergent differentiation pathways. We therefore decided to include explicit lineage information and analyse lineage relationships in a high-throughput manner using the LINNAEUS method^24^. In LINNAEUS, cells are marked by heritable DNA barcodes (genetic scars) that are created by Cas9 during early development (Methods) and whose distinct sequences identify cells that descended from the same parent at the time the scars were created. Here, we injected Cas9 into one-cell-stage embryos in order to record lineage relationships during early development until gastrulation^24^ (Fig. 1a), which we read out later in the adult heart (all lineage trees are shown in Supplementary Figs. 14–17). By sequencing scars and transcriptomes from the same single cells, we could build lineage trees that revealed the shared developmental origins of the cell types (Fig. 4a and Methods).

We first validated this approach in the adult heart using two control experiments. As expected, when preparing separate single-cell suspensions for the atrium and ventricle, the lineage tree showed separate atrial and ventricular branches, whereas a randomly split suspension did not show branch separation (Supplementary Fig. 18). We next sought to integrate the data across biological replicates in order to infer reproducible lineage relationships between cell types. In a lineage tree, all cells in a node share the same developmental ancestor, and any transient cell originating from a cell in a node can be found in the same node. We calculated correlations between cell type ratios in the different tree nodes to determine which cell types were related by lineage (Fig. 4b and Methods). Hierarchical clustering of these correlations revealed four clusters of cell types at 3 d.p.i. and seven clusters at 7 d.p.i. (Fig. 4c,d, Supplementary Fig. 19 and Methods). At both time points, all immune cells shared a common lineage, validating our approach. We observed a clustering of several fibroblast types, including *col11a1a*, *col12a1a* and constitutive fibroblasts, together with epicardial cells, strongly suggesting that these fibroblast cell types share a developmental origin with the epicardium (Fig. 4c,d).

To validate the lineage origins of *col11a1a* and *col12a1a* fibroblasts, we performed a genetic Cre–*lox* lineage tracing experiment in regenerating hearts using the transgenic line *TgBAC(tcf21:Cre-ERT2; ubi:Switch)*. Our scRNA-seq data showed that *tcf21* was expressed in epicardial cells, constitutive fibroblasts and other fibroblast types of the epicardial cluster (Supplementary Fig. 20). After recombination, expressed mCherry at 7 d.p.i. colocalized with endogenously expressed *col12a1a* (Fig. 4e), corroborating the origin of the transient *col11a1a* and *col12a1a* fibroblasts as being the epicardium or epicardial-derived fibroblasts.

LINNAEUS reliably identifies the developmental origins of cell types, but lineage recording is limited to early development. It therefore remained unclear from which source cell type the transient fibroblasts originated following injury in the adult heart – for instance, we could not distinguish whether *col11a1a* and *col12a1a* fibroblasts were derived from epicardial cells, constitutive fibroblasts or any other cell type in the epicardial cluster. Furthermore, expression of *tcf21* is not specific enough to address this question using Cre–*lox* lineage tracing (Supplementary Fig. 20), and reporter-based approaches reach their limits when analysing cell states with high resolution (our data did not identify suitable marker genes that could be used for lineage tracing of constitutive fibroblasts). To further elucidate the origins of the transient epicardial fibroblast states, we therefore applied a transcriptome-based trajectory inference method based on PAGA and RNA velocity^39^ to all ventricular cell types from epicardial lineage clusters at 3 d.p.i. and 7 d.p.i. (Fig. 4f, Supplementary Fig. 21 and Methods). At both time points, this approach showed that transient *col11a1a* and *col12a1a* fibroblasts originated from constitutive fibroblasts (Fig. 4f and Supplementary Fig. 21a) and suggested that the extremely transient *col11a1a* fibroblasts (which were only present at 3 d.p.i.) turned into *col12a1a* fibroblasts at 7 d.p.i. Genes that were upregulated along these trajectories were those encoding collagens *col11a1a* and *col12a1a*, proregenerative ECM factor *fn1a*, epicardial activation marker *postnb*, retinoic acid signalling response gene *stra6* and cardiomyocyte mitogen *nrg1* (Supplementary Fig. 21b,c).

### Identification of endocardial fibroblasts

**Fig. 5:**
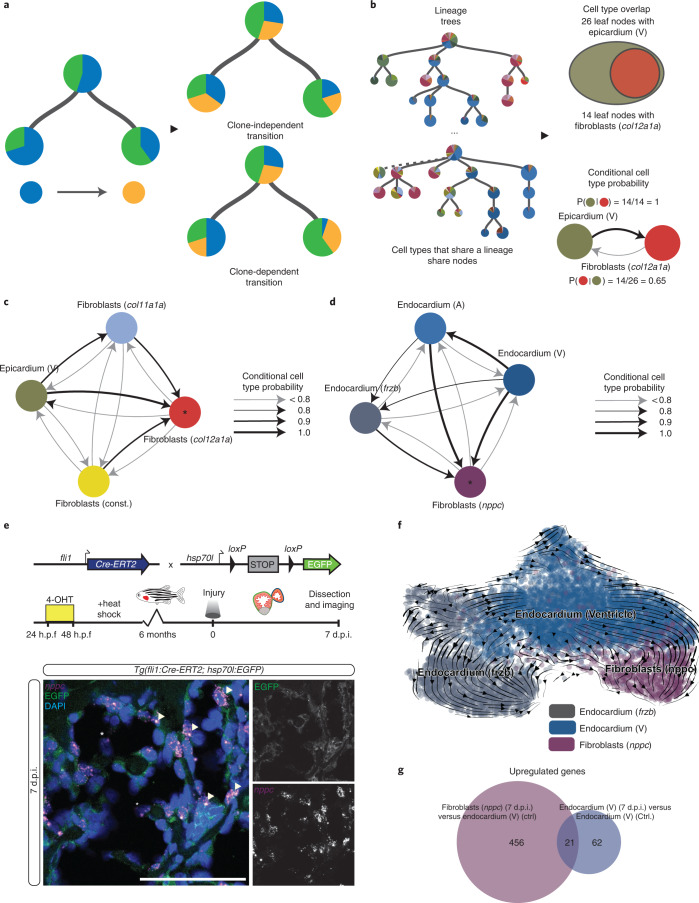
Identification of endocardial fibroblasts. **a**, Clone-independent transitions (for example 50%, top) showed similar transition rates across clones, leading to a strong correlation between yellow and blue cell types. Clone-dependent transition rates (for example 30% and 80%, bottom) caused yellow and blue cell types to lose correlation, requiring a different approach for lineage analysis. **b**, Conditional cell type probabilities could pinpoint lineage origins despite clone-dependent transitions. **c**, Conditional probabilities reproduced the epicardial lineage origin of *col12a1a* fibroblasts. **d**, Endocardial cell types were present in more than 80% of all nodes containing *nppc*, *spock3* and valve fibroblasts. Asterisks in **c** and **d** indicate the queried cell type. **e**, Cre–*lox* lineage tracing confirmed the endocardial origin of *nppc*-expressing fibroblasts in cryoinjured hearts at 7 d.p.i. Sections were stained for GFP (green) and *nppc* (magenta). Arrowheads point to *nppc* and EGFP colocalization. Asterisks point to *nppc*-positive delaminating cells. Scale bar, 100 μm. **f**, Trajectory analysis revealed a potential transition from endocardial cells to *nppc* fibroblasts. **g**, Venn diagram of upregulated genes in *nppc* fibroblasts compared with activated endocardium (7 d.p.i.). Source data

The transient *nppc* fibroblasts and several other fibroblast subtypes (*spock3*, *cfd* and valve fibroblasts) were not part of the epicardial lineage cluster at 3 d.p.i. or 7 d.p.i. but displayed moderate positive correlations with the endocardium and with each other. We reasoned that our correlation-based analysis had an underlying assumption that all clones would exhibit similar transition rates (Fig. 5a). This assumption may not be true for all cell types, as clones located in the uninjured part of the heart may not respond to the perturbation. When downsampling to 50% of the clones, we found that the cluster of epicardial fibroblasts remained stable, whereas the correlations among *nppc*, *spock3*, *cfd* and valve fibroblasts varied between iterations (Supplementary Fig. 22 and Methods). Such variability would be expected if there were considerable differences between the activation profiles of the individual clones that make up these cell types.

We therefore developed a second computational approach, in which we analysed the conditional probabilities of co-occurrence of a cell type of interest with all other cell types (Fig. 5b and Methods). We reasoned that such a targeted approach would yield more reliable results specifically for cell populations of variable clonal behaviour. As validation, we found that *col11a1a* fibroblasts, constitutive fibroblasts and epicardium (ventricle) were present in >90% of the 3 d.p.i. lineage tree nodes that contained *col12a1a* fibroblasts (Fig. 5c). At 7 d.p.i., we found that the different endocardial cell types (endocardium (atrium), endocardium (ventricle) and endocardium (*frzb*)) were present in more than 80% of all nodes containing transient *nppc* fibroblasts, suggesting a lineage relationship between this fibroblast type and the endocardium (Fig. 5d). Similarly, the analysis suggested that *spock3* and valve fibroblasts were also of endocardial origin, whereas *cfd* fibroblasts were of epicardial origin (Supplementary Fig. 23). Hence, our analysis revealed the existence of three endocardial-derived fibroblast subtypes (*nppc*, *spock3* and valve fibroblasts), whereas all other fibroblasts shared a lineage origin with the epicardium. To validate our computational approach, we performed genetic lineage tracing using the transgenic line *Tg(fli1:Cre-ERT2; hsp70l:Switch)*, which, after embryonic recombination, marks endothelial cells in the adult heart^14,17^. Expressed EGFP at 7 d.p.i. colocalized with endogenously expressed *nppc* (Fig. 5e), corroborating the endocardial origin of the transient *nppc* fibroblasts.

Only ~20% of the nodes with endocardium (ventricle) contained *nppc* fibroblasts (Supplementary Fig. 24a), suggesting that endocardial fibroblasts are only generated in a subset of the endocardium, potentially in the injury area. This clone-specific injury reaction of the endocardium would explain why targeted lineage analysis based on conditional probabilities outperforms correlation-based lineage analysis. We hypothesized that the internal position of the endocardium, as well as the transient nature and low clonality of the *nppc* fibroblasts, might mean that these cells are only generated from damaged endocardial clones. We confirmed that longer contact of the cryoprobe with the heart led to a larger injury area and resulted in much stronger *nppc* expression beyond the border zone, compared with injuries resulting from shorter contact times (Supplementary Fig. 24b,c and Methods).

Trajectory analysis revealed transcriptional similarity and a potential transition path between ventricular endocardium and *nppc* fibroblasts (Fig. 5f). We observed that *nppc* fibroblasts continued to express endothelial genes (for example *vwf* and *fli1a*) in addition to ECM genes, suggesting that they maintain at least part of their endocardial gene expression after turning on a fibrotic gene expression program. This prompted us to investigate in more detail to what degree the gene expression profile of these cells had fibroblast characteristics. In particular, we considered whether the *nppc* fibroblasts might correspond to the activated endocardium that was previously described after injury^13^. We observed from our single-cell transcriptomic data that the endocardium underwent an activation response at 7 d.p.i. compared with healthy control hearts (Fig. 5g). However, this response was weaker than and only partially overlapping with the gene expression profile of *nppc* fibroblasts. Hence, the endocardium responds to injury with two separate transcriptional responses, which differ in the magnitude of their expression changes and which have not been systematically distinguished in the previous literature. Differentially expressed genes of *nppc* fibroblasts include many ECM-related factors (which led us to identify this cluster as a fibroblast state as shown in Fig. 2c) (Supplementary Data File 3). Furthermore, we noticed that *nppc* fibroblasts had upregulation of *twist1b* and express *snail2*, both of which are classical markers of epithelial-to-mesenchymal transition. This, together with the observation that some *nppc*^+^ cells appeared to be delaminating in our microscopy images (Fig. 5e), suggested that *nppc* fibroblasts may undergo at least a partial transition toward a mesenchymal state. However, as we did not observe a clear downregulation of VE-cadherin (*cdh5*) or upregulation of N-cadherin (*cdh2*), we speculate that *nppc* fibroblasts do not acquire full mesenchymal characteristics. Of note, it was previously reported that endocardial cells express collagen following injury but do not undergo a complete epithelial-to-mesenchymal transition^17^, whereas another publication showed that a minor proportion of endocardial cells appear to undergo a more complete transition^14^.

In summary, our analyses revealed a clear separation of epicardial- and endocardial-derived fibroblasts, with both lineages giving rise to distinct transient fibroblast states following injury. An epicardial origin for cardiac fibroblasts has also recently been established in zebrafish^14,40,41^, and our analysis provides strong evidence for the existence of fibroblasts of endocardial origin in the atrium and ventricle, as previously suggested in mouse^42,43^ and zebrafish^14^.

### Cellular dissection of the role of canonical Wnt signalling

**Fig. 6:**
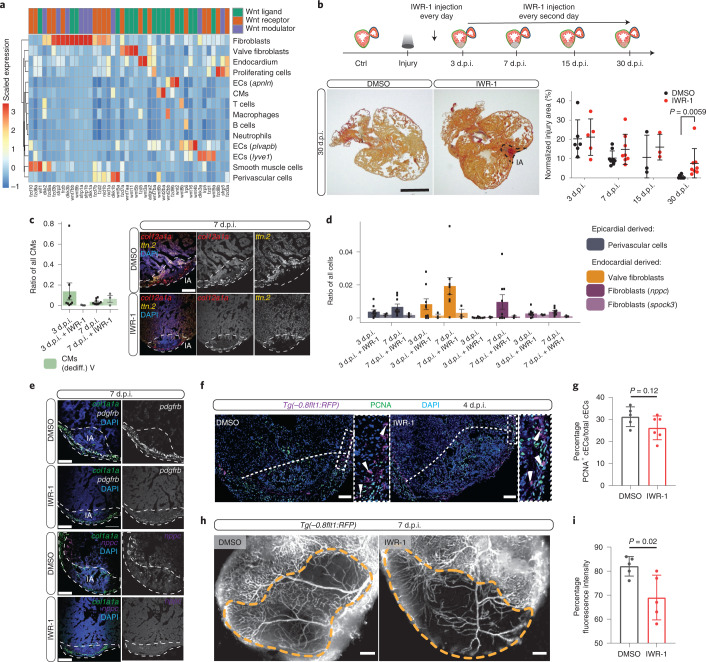
Cellular dissection of the role of canonical Wnt signalling. **a**, Expression of Wnt signalling factors in different cell types of the zebrafish regenerating heart. **b**, Upper panel: cartoon summary of IWR-1 Wnt inhibition experiments. Lower left: histological comparison of the IA at 30 d.p.i. with intraperitoneal (IP) injections of IWR-1 or DMSO. In the paraffin AFOG-stained sections, fibrin-red and collagen-blue are clearly visible in the IA in IWR-1-treated samples at 30 d.p.i. Scale bar, 300 μm. Lower right: relative size of the IA area as a percentage across all histological replicates; data shown as mean and s.d. Two-tailed unpaired Student’s *t* test between groups at each time point, *P* = 0.0059. **c**, Left: changes in relative numbers of dedifferentiated CMs at 3 and 7 d.p.i. between IWR-1- and DMSO-treated hearts (error bars indicate s.e.m.). Right: localization of dedifferentiated (*ttn.2*) cardiomyocytes at 7 d.p.i. with and without Wnt inhibition. Scale bar, 100 μm. **d**, Changes in relative numbers of non-CMs following Wnt inhibition at 3 and 7 d.p.i. (error bars indicate s.e.m.). **e**, Fluorescence in situ hybridization of perivascular cells (*pdgfrb*, white) and *nppc* fibroblasts (*nppc*, purple) at 7 d.p.i. with and without Wnt inhibition. Scale bar in **c** and **e**, 100 μm. White dashed lines indicate the IA. **f**, Immunostaining of *Tg(-0.8flt1:RFP)* hearts at 4 d.p.i. with and without Wnt inhibition; RFP (coronaries, magenta), PCNA (proliferation marker, green) and DNA (DAPI, blue). Arrowheads point to PCNA^+^ cECs, white dashed lines indicate the IA. Scale bar, 100 μm. **g**, Percentages of PCNA^+^ cECs in DMSO-injected (*n* = 5) and IWR-1-injected (*n* = 6) fish at 4 d.p.i. Data are shown as mean and s.d. Two-tailed unpaired Student’s *t* test, *P* = 0.12. **h**, Whole-mount images of *Tg(-0.8flt1:RFP)* hearts at 7 d.p.i. with and without Wnt inhibition. Orange dashed lines indicate injury area. Scale bar, 100 μm. **i**, Percentages of RFP fluorescence intensity in injured tissue of DMSO-injected (*n* = 5) and IWR-1-injected (*n* = 5) fish at 7 d.p.i. Data are shown as mean and s.d. Two-tailed unpaired Student’s *t* test, *P* = 0.02. Source data

We noticed that fibroblasts expressed many genes related to Wnt signalling (ligands, receptors and modulators) (Fig. 6a and Supplementary Fig. 25), which inspired us to investigate the role of canonical Wnt signalling in this system. The role of Wnt signalling in heart regeneration remains an important open question^44^. On the one hand, Wnt is generally considered to be a proproliferative factor, and Wnt activation has been shown to be beneficial for zebrafish fin and spinal cord regeneration^33,45^. On the other hand, the role of Wnt signalling in cardiomyocyte dedifferentiation and proliferation is controversial^46–48^. We hypothesized that Wnt might exert its complex functions by regulating activation of proregenerative cell states, and we investigated whether cells with a shared lineage origin exhibited similar changes following a stimulus, that is, whether lineage relationship is predictive of perturbation response.

We therefore inhibited canonical Wnt signalling after cryoinjury using the well-characterized Wnt/β-catenin-dependent signalling inhibitor IWR-1 and observed the effects at 3, 7, 15 and 30 d.p.i. (Fig. 6b and Methods). Wnt/β-catenin signalling inhibition led to a significant delay in heart regeneration, with prolonged fibrosis and increased size of injury area compared with the control (Fig. 6b). Single-cell transcriptomics of IWR-1-treated hearts at 3 d.p.i. and 7 d.p.i. revealed that cardiomyocyte dedifferentiation was delayed (Fig. 6c). Compared with control samples, numbers of dedifferentiated cardiomyocytes were reduced at 3 d.p.i., and they did not localize to the injury area at 7 d.p.i. (Fig. 6c).

Levels of perivascular cells and all endocardial fibroblasts (*nppc*, *spock3* and valve fibroblasts) were strongly reduced after Wnt inhibition, whereas the other transiently increased fibroblasts remained at similar levels overall (Fig. 6d, Supplementary Fig. 26 and Supplementary Data File 4). Specifically, the overall abundance of *col11a1a* and *col12a1a* fibroblasts did not change (Supplementary Fig. 26), even though *col12a1a* fibroblast numbers peaked earlier after Wnt inhibition (at 3 d.p.i. instead of 7 d.p.i.); this may have been an indirect effect due to the observed impairment of heart regeneration. We confirmed these findings by fluorescence in situ hybridization (Fig. 6e). These observations strongly suggest that Wnt signalling is required for the activation of endocardial fibroblasts, akin to the described role of Wnt in inducing endothelial-to-mesenchymal transition in mice^49,50^.

We next wanted to understand how the absence of these cell types could mediate the observed delay in regeneration. Perivascular cells are known to exert a proangiogenic function through signalling and mechanical stabilization of nascent vasculature^51^. High expression of the chemokine *cxcl12b* in perivascular cells in our data set (Fig. 3c) further supported the putative proangiogenic role of these cells. Furthermore, NPPC signalling has been shown to have a complex range of functions in the cardiovascular system^52,53^ and has recently been suggested to be involved in fin regeneration^54^. We therefore hypothesized that Wnt inhibition might delay heart regeneration via reduced revascularization following injury.

To assess revascularization, we quantified the proliferation of coronary endothelial cells (cECs) at 4 d.p.i. as well as the coverage of coronaries in the injury area at 7 d.p.i. (Methods). Following administration of IWR-1, we observed a marginal but not significant reduction in cEC proliferation at 4 d.p.i. (Fig. 6f,g). However, at 7 d.p.i., we observed a clear and statistically significant reduction in the coverage of coronary vessels in the injury area (Fig. 6h,i). In summary, these results suggest that the observed reduction in heart regeneration following Wnt inhibition is at least partially mediated by a revascularization defect. This defect does not appear to be caused by reduced proliferation of cECs and might instead be linked to other aspects of revascularization, such as migration of coronaries or interactions with perivascular cells.

## Discussion

Here, we used a combination of single-cell transcriptomics and spatiotemporal information to characterize putative proregenerative cell states in the adult zebrafish heart in a data-driven manner. This approach allowed us to identify three transient cell states with fibroblast characteristics (*col11a1a*, *col12a1a* and *nppc* fibroblasts) that are major sources of known proregenerative genes and also express additional secreted factors with potential proregenerative functions. These fibroblast cell states are ideally suited to be cellular drivers for regeneration based on their expression profiles, their location in the injury area and their transient appearance after injury. By combining high-throughput lineage tracing with RNA velocity and single-cell trajectory inference, we were able to systematically identify the origin of these cell states. This approach effectively combines information about two time points – CRISPR barcoding during early development and trajectory inference at the time of analysis. Furthermore, we developed two complementary computational approaches for integrating CRISPR barcoding data across biological replicates in order to obtain statistically sound information on lineage relationships between cell types. Taken together, the results obtained with these approaches can serve as a blueprint for understanding the origin of transient cell states that are generated under disease conditions.

Several lines of evidence (CRISPR lineage analysis, Cre–*lox* lineage tracing, transcriptome profiles and long versus short injury) showed that *nppc* fibroblasts were derived from the endocardium. The endocardium exhibited two separate activation programs that had not previously been systematically distinguished: *nppc* fibroblasts and a less pronounced endocardial activation profile. Functional experiments showed that activation of endocardial fibroblasts depends on canonical Wnt signalling. Wnt inhibition leads to a delay in regeneration which is accompanied by a revascularization defect. Although it is tempting to link the observed depletion of *nppc* fibroblasts and perivascular cells following Wnt inhibition to reduced revascularization, further experiments would be needed to substantiate this point. A combination of CRISPR lineage tracing, trajectory inference and Cre–*lox* lineage tracing showed that the *col11a1a* and *col12a1a* fibroblasts shared their lineage origin with the epicardium and were generated from constitutive fibroblasts following injury. *Col11a1a* and *col12a1a* fibroblasts have very similar transcriptional profiles, and our RNA velocity analysis suggested that *col11a1a* fibroblasts are precursors of *col12a1a* fibroblasts. Targeted cell type depletion of *col12a1a*-expressing cells after injury led to impaired heart regeneration, supporting the proregenerative function of these cells. In summary, our detailed cellular dissection of the fibrotic response in the zebrafish heart characterized sources and mechanisms of proregenerative programs. The large diversity of fibroblast cell populations found in the regenerating zebrafish heart suggests that fibrosis should be seen as a collective phenomenon to which a variety of cell types and states contribute.

The pathways that contribute to the regenerative capacity of the zebrafish heart are conserved in humans. Thus, our analysis may lead us to better understand the limited regenerative capacity of the human heart, while at the same time opening up an exciting strategy to identify potential therapeutic approaches. Our data set and the experimental and computational approaches presented here will serve as a powerful resource for identification of additional candidate interactions between cell states involved in heart regeneration.

## Methods

### Ethics statement

This study complied with all relevant ethical regulations. Zebrafish were bred, raised and maintained in accordance with the FELASA guidelines^55^, the guidelines of the Max Delbrück Center for Molecular Medicine and the Max Planck Society, and the local authorities for animal protection (Landesamt für Gesundheit und Soziales, Berlin, and The Veterinary department of the Regional Board of Darmstadt, Darmstadt, Germany) for the use of laboratory animals, based on the current version of German law on the protection of animals and EU directive 2010/63/EU on the protection of animals used for scientific purposes. In addition, housing and breeding standards followed the international ‘Principles of Laboratory Animal Care’ (NIH publication no. 86-23, revised 1985).

### Zebrafish

Experiments in this study used the zebrafish wild-type strain *AB* and transgenic lines *Tg[ubi:zebrabow-M]*^*a131Tg*^ (ref. ^27^), *TgBAC[cryaa:EGFP, tcf21:Cre-ERT2]*^*pd4*^ (ref. ^41^), *Tg[-3.5ubi-loxP-EGFP-loxP-mCherry] (ubi:Switch)*^56^, *Tg(fli1:Cre-ERT2)*^*cn9*^ (ref. ^17^), *Tg(–1.5hsp70l:loxP-STOP-loxP-EGFP, cryaa:Venus)*^*zh701*^ (ref. ^57^), *TgUAS:NTR-mCherry)*^58^, *Tg(-0.8flt1:RFP)*^*hu5333*^ (ref. ^59^) and *Tg(-4kbcol12a1a:GAL4VP16, ins:YFP)* (this study). Adult zebrafish of random sex, aged between 4 months and 1 year and with a length of at least 3 cm were used in all experiments. Animals were randomly assigned to the test groups in all experiments.

### Cryoinjury procedure

Cryoinjury was performed as previously described^60,61^. Briefly, fish were presedated in water containing 0.03 mg ml^−1^ Tricaine (PHARMAQ, pH 7). The concentration was then increased to 0.168 mg ml^−1^ for anesthesia. Fish were placed with the ventral side facing up in a foam holder under a dissecting scope. To access the heart, a small incision was made through the body wall and the pericardium, using microdissection forceps and scissors. Once the pericardial sac was opened, the heart ventricle was exposed by gently compressing the abdomen. Excess water was carefully removed by blotting with tissue paper, not allowing the fish skin to dry. Then, a stainless steel cryoprobe precooled in liquid nitrogen was applied to the ventricular wall for 20 s. Fish were then placed in a tank of fresh system water with 1.5 mg l^−1^ morphine sulfate for 6 h^62^; for the analysis of scRNA-seq data presented here, we also included two morphine-treated and two control hearts from Lelek et al.^62^ (~20,000 cells, about 10% of the total data set). Reanimation was enhanced by gill oxygenation, in which water around the gills was aerated by pipetting for a couple of minutes. To investigate the effects of the cryoinjury depth, fish were injured using the same procedure, but the cryoprobe was applied for 5 s, 10 s, 15 s, 20 s and 25 s, respectively. Established protocols use 20 s of cryoprobe application^60,61^. For all injuries, a timer was used to assure the reproducible timing of the cryoprobe contact with the heart tissue. Hearts were analysed at 3, 7, 15 and 30 d.p.i.

### Cas9 and sgRNA injections for high-throughput lineage tracing

For CRISPR–Cas9 lineage tracing, we used the same experimental approach as previously described^24^. In brief, we set up crosses between zebrabow-M^27^ adults with high dTomato fluorescence, and we injected the embryos at the one-cell stage with 2 nl Cas9 protein (NEB, final concentration 350 ng µl^−1^) in combination with an sgRNA targeting dTomato (final concentration 50 ng µl^−1^, sequence: GGTGTCCACGTAGTAGTAGCGTTTTAGAGCTAGAAATAGCAAGTTAAAATAAGGCTAGTCCGTTATCAACTTGAAAAAGTGGCACCGAGTCGGTGCTTTT). Loss of dTomato fluorescence was used as a marker of successful generation of genetic scars. Injected fish were raised to adulthood for scRNA-seq analysis of the heart.

### Preparation of single-cell suspensions

Adult zebrafish (injured and uninjured) were humanely killed by immersion in ice-cold water (0–4 °C) for 20 min. The heart was dissected from the fish and transferred into cold Hanks’ Balanced Salt Solution (HBSS). The dissection included the atrium, ventricle and bulbus arteriosus, except for samples in which only the atrium or the ventricle was isolated. A needle and a syringe filled with cold HBSS were used to pierce the lumen of the heart and thoroughly wash away most of the erythrocytes. Afterwards, the heart was opened carefully with forceps, and the tissue was incubated at 37 °C for 30 min in 500 µl HBSS containing Liberase enzyme mix (Sigma-Aldrich, 0.26 U m^−1^ final concentration) and Pluronic F-68 (Thermo Fisher Scientific, 0.1%) while shaking at 750 r.p.m. on an Eppendorf ThermoMixer F2.0 with intermittent pipette mixing. After most of the tissue had been dissociated, the reaction was stopped by adding 500 µl cold HBSS supplemented with 1% bovine serum albumin (BSA). The suspension was centrifuged at 250*g* at 4 °C and washed two times with 500 µl cold HBSS containing 0.05% BSA, then filtered through a cell strainer of 35 µm diameter. The quality of the single-cell suspension was then confirmed under a microscope, and cells were counted before scRNA-seq library preparation. We noticed some fluctuations in cell type ratios detected by scRNA-seq (Figs. 2a and 3a), which we speculate could have been due to variability in the cryoinjury procedure or in the timing of the repair process. Furthermore, our dissociation protocol might have led to a depletion of cardiomyocytes, which, as they are large cells, tend to break relatively easily. Indeed, cardiomyocytes on average have lower transcript counts than nonmyocytes (~1,600 compared with ~4,500 transcripts per cell), which may indicate that we detected nuclei rather than cells in the case of cardiomyocytes.

### Single-cell RNA-seq and scar detection

Single cells were captured using a Chromium Single Cell 3′ kit (10X Genomics, PN-1000075), according to the manufacturer’s recommendations. We aimed for 10,000 cells per library whenever possible. Both v2- and v3-chemistry were used for the data presented here. For simultaneous detection of transcriptome and CRISPR-induced genetic scar sequences for lineage tracing, we proceeded as previously described^24^. In brief, the scar sequences were enriched by a two-round nested PCR approach, using 10 µl of the 10X Genomics cDNA and target site specific primers (dTomato transgene). The scar library was then indexed using the indexing primers of the Chromium kit to ensure they could be sequenced and demultiplexed together with the transcriptome. Samples were sequenced on an Illumina NextSeq 500 150 bp and an Illumina HiSeq 2500 200 bp after successful quality control by Bioanalyzer (DNA HS kit, Agilent).

### Mapping and clustering of single-cell mRNA data

A zebrafish transcriptome was created with Cell Ranger 3.0.2 from GRCz11, release 92. Alignment and transcript counting of libraries were done using Cell Ranger 3.0.2. Library statistics are summarized in Supplementary Data File 1. The transcriptome data were filtered, clustered and visualized using Seurat 3.0 (ref. ^63^). We excluded the cluster of *mpeg1.1* fibroblasts (Fig. 2c,d) from further analysis, as we could not rule out the possibility that the expression of the macrophage marker *mpeg1.1* in this fibroblast cluster was an artifact caused by, for example, fragmentation of macrophages^64^.

### Histological staining, analysis and imaging

For the analysis of the injury areas, animals were humanely killed at different times after injury by placing them in ice-cold water at 0–4 °C for 20 min. Hearts were dissected and incubated in 2 U ml^−1^ heparin and 0.1 M KCl in phosphate-buffered saline (PBS) for around 30 min. Cryosamples were fixed in 4% paraformaldehyde (PFA) in PBS overnight at 4 °C, washed in PBS for 3× 10 min and incubated overnight in 30% sucrose in PBS. Samples were then frozen in Tissue-Tek O.C.T Compound (Sakura) on dry ice. Tissue was cut at 7 μm on a cryostat (Leica) using Superfrost slides (Thermo Fisher Scientific). For paraffin sections, tissue was fixed in 4% PFA in PBS overnight at 4 °C, washed in PBS for 3× 10 min and incubated in an ethanol series at 30% for 10 min, 50% for 20 min and 70% overnight at 4 °C. Next, samples were dehydrated in an ethanol series at 80% for 30 min, 90% for 30 min, 96% for 1 h, 100% for 5 min and 100% for 1 h, then in Toluol for 45 min and Toluol for 15 min, and finally embedded in paraffin. Connective tissue was stained using acid fuchsin orange G (AFOG). In brief, slides were dried for 30 min at room temperature. Next, slides were incubated in Bouin Solution (Sigma-Aldrich) for 2 h at 60 °C and left for overnight incubation under the hood. Slides were washed for 30 min under running double-distilled water (ddH_2_O) and incubated for 7 min in 1% phosphomolybdic acid (Sigma-Aldrich). Samples were washed for 3 min in running ddH_2_O and incubated with AFOG solution (made in our laboratory, Sigma-Aldrich) for 3 min. Slides were washed until clear with running ddH_2_O and rehydrated with 70%, 94% and 2× 100% ethanol and for 2× 5 min with xylol (Sigma-Aldrich). Slides were mounted with xylene mounting medium (Merck) and allowed to dry overnight under the hood. For the analysis of injury size, the total ventricular tissue area and injury area on multiple slides were measured. Injury area was identified by visualization of collagen (blue) and fibrin (red). Images were obtained with a Keyence Microscope BZX800 using Viewer and analysed with ImageJ/Fiji (v.2.1.0).

### RNAscope Multiplex Fluorescent v2 assay for immunofluorescence in situ hybridization and imaging

The RNAscope assay (ACD) for fluorescence in situ hybridization was used to localize different messenger RNAs (mRNAs) expressed by various cell types during heart regeneration. The technique was performed according to the manufacturer’s instructions for fixed frozen tissues (323100-USM) or in combination with antibody staining (MK 51-150). Cryosections (7 µm) were dried for 30 min at room temperature before the experiment. The following changes were made: slides were incubated at 99 °C for 15 min with an antigen retrieval solution (ACD) using a steamer (WMH). Signal development was done using Tyramide Signal Amplification (TSA) plus fluorophores (PerkinElmer) fluorescein, cyanine 3 and cyanine 5 at 1:1,000 dilution. The probes used are listed in Supplementary Table 1. In addition, anti-EGFP (Sigma-Aldrich, catalog no. G1544, 1:500) and secondary antibody AlexaFluor 488 goat anti-rabbit IgG (Thermo Scientific, catalog no. A-11008, 1:500) were used in the protocol combining immunohistochemistry with in situ hybridization. The fluorescence in situ hybridization for the whole-mount embryo was performed according to the manufacturer’s instructions (MK 50-016). Images were obtained with a Zeiss LSM880 confocal microscope using Zen 2.3 SP1 FP3 (v.14.0.0.0) software and analysed using ImageJ/Fiji (v.2.1.0) and Photoshop (v.20.0.6) software.

### Microscopy analysis of cardiomyocyte proliferation

Zebrafish hearts were extracted and fixed in 4% PFA in PBS overnight, washed in PBS for 3× 10 min and incubated overnight in 30% sucrose in PBS. Samples were then frozen in Tissue-Tek O.C.T. Compound (Sakura) on dry ice. Tissues were cut at 7 μm on a cryostat (Leica) using Superfrost slides (Thermo Fisher Scientific). For immunofluorescence, samples were washed with PBST (1× PBS, 0.1% Triton X-100) before permeabilization with Antigen Unmasking Solution (Vector Laboratories, Inc. (H-3300), 1:100) at 80 °C for 20 min. Samples were then washed twice with PBST and incubated in blocking solution (3% BSA, 5% goat serum, 0.2% Triton X-100 in PBS), then incubated with primary antibodies overnight at 4 °C, followed by two PBST washes and incubation with secondary antibodies for 2 h at room temperature. Slides were washed again with PBST and mounted with mounting medium Vectashield with DAPI (Vector Laboratories, Inc.). The primary antibodies used were anti-Mef2C (Santa Cruz Biotechnology, catalog no. sc-313, 1:200) and anti-PCNA (Dako, catalog no. M0879, 1:500). The secondary antibodies were AlexaFluor 488 goat anti-rabbit IgG (Thermo Scientific, catalog no. A-11008, 1:500) and AlexaFluor 633 goat anti-mouse IgG (Thermo Scientific, catalog no. A-21050, 1:500). Confocal images were taken using an LSM880 microscope (Zeiss). The percentage of proliferating cardiomyocytes was calculated as a ratio of the number of (PCNA + Mef2C)^+^ particles to the total number of Mef2c^+^ particles in the injury area and in 200 μm of the injury border zone using ImageJ (v.2.1.0). Briefly, the total number of cardiomyocytes was analysed in the Mef2c^+^ channel, as the total number of particles that were segmented and counted using the particle analysis tool after thresholding and applying a despeckling filter. The number of (PCNA + Mef2C)^+^ particles was determined using the particle analysis tool after merging the green and red (identified using look-up tables) channels and selecting a colour threshold for yellow. Thresholding was verified visually using the original image. For each biological replicate, three nonconsecutive midsagittal sections were used.

### Microscopy analysis of revascularization

Zebrafish ventricles were extracted from adults that had not been treated with morphine and fixed in 4% PFA for 1 h at room temperature. Ventricles were embedded in OCT (Tissue-Tek) and sectioned (8 µm). For each biological replicate, three nonconsecutive midsagittal sections were used. Immunostaining was performed as previously described^65^. The primary antibodies used were anti-RFP (Rockland, catalog no. 600-401-379, 1:200) and anti-PCNA (Santa Cruz Biotechnology, catalog no. sc-56, 1:500). The secondary antibodies were AlexaFluor 488 goat anti-mouse IgG (H+L) (Thermo Scientific, catalog no. A-11029, 1:500) and AlexaFluor 647 goat anti-rabbit IgG (H+L) (Thermo Scientific, catalog no. A-21244, 1:500). Confocal images were obtained using an LSM700 microscope (Zeiss). The percentage of proliferating cECs (PCNA^+^) was calculated as the ratio of the total numbers of cECs in the injury area and in 200 µm of the injury border zone using ZEN 3.2 Blue software. For fluorescence intensity analysis, the percentage fluorescence was calculated from whole-mount images as a ratio to the background fluorescence in the injury area using Fiji software.

### Wnt inhibition

To investigate the role of Wnt signalling in heart regeneration, the Wnt antagonist IWR-1-endo (Sigma-Aldrich) was used^46,66^. IWR-1 was dissolved in dimethyl sulfoxide (DMSO) to prepare a 10 mM stock solution. Wild-type fish were injected intraperitoneally with 25 μl of 10 μM IWR-1 in PBS or DMSO (0.1% in PBS)^67^. The injection was administered at 1 d.p.i. and 2 d.p.i. for the 3 d.p.i. analysis and, for later time points, once every 2 days from 2 d.p.i. until the day fish were euthanized.

### Cloning and transgenesis of *-4kbcol12a1a*:GAL4VP16

The possible 5′-upstream promoter region upstream of the predicted transcription start site of *col12a1a* was identified using *Danio rerio* strain Tuebingen chromosome 17, GRCz11 Primary Assembly NCBI Reference Sequence: NC_007128.7, and served as a target template for primer design. Forward (5′-GAGAGAGAGAAAGCACCATTCTG-3′) and reverse (5′-GTTTACACACACACAGTCAGCAG-3′) primers were designed using plasmid editor ApE and used to amplify the promoter region by PCR from genomic DNA of *D. rerio*. PCR was carried out in a 25 µl reaction using GoTaq Long PCR Mastermix (Promega, cat no. M4021) according to the manufacturer’s protocol and TOPO cloned into pENTR5′ (Invitrogen) to create pAM222. *Tg(-4kbcol12a1a:GAL4VP16)* is a multisite gateway assembly of *pAM222*, Tol2kit no. 387 *(pME- GAL4VP16)*, no. 302 (*p3E_SV40polyA*) and pAM57 (*pDestTol2 insulin:YFP*). To obtain pAM57, the *crystallin* promoter was exchanged with the minimal *insulin* promoter. First, using site-directed mutagenesis, pAM58, an empty pDestTol2 vector containing YFP with an added restriction side for insertion of any promoter of interest, was generated. Deletion primers were engineered by designing standard, nonmutagenic forward and reverse primers that flanked the region to be deleted (5′-GGTACCGTAAAACGACGGCCAGTGAATTATC-3′ and 5′-GATATCATGGTGAGCAAGGGCGAGGAGCTGTTC-3′, respectively). In the forward primer, the sequence for the restriction site EcoRV was included. The minimal *insulin* promoter was amplified from genomic DNA and inserted using In-Fusion Cloning technology (Takara). The primers were designed using the In-Fusion Cloning Primer Design Tool (www.takarabio.com): minimal *insulin* promoter forward primer 5′-TTTTACGGTACCGATCTTCAGCCCACAGTCTAGTTTAG-3′ and reverse primer 5′-CTTGCTCACCATGATCGAAGCAGAGGCGAGGAATG-3′. PCR was carried out in a 25 µl reaction using Clone Amp HiFi PCR premix. The In-Fusion HD cloning reaction was performed with cleaned products of *pDestTol2 GATATC-YFP* (*EcoRV*-digested) and minimal *insulin* promoter PCR product using In-Fusion HD enzyme premix. The minimal *insulin* promoter is ideal as a selection transgenesis marker because of its small size and very early activity (from 24 h postfertilization; h.p.f.).

For Tol2-mediated zebrafish transgenesis, 25 ng µl^−1^ Tol2 mRNA was injected with 25 ng µl^−1^ plasmid DNA. F0 founders were screened for specific *insulin*:YFP expression, raised to adulthood and screened for germline transmission. The activity of the isolated 4-kb upstream region of zebrafish *col12a1a* corresponded to that of the endogenous *col12a1a* expressed in embryos of various stages (Supplementary Fig. 12a). In uninjured adult hearts, RFP expression in *col12a1a*>NTR:RFP-labelled cells colocalized with endogenous *col12a1a* expressed in the epicardium (Supplementary Fig. 12b). As expected, cryoinjury transiently induced both endogenous *col12a1a* and RFP expression in the injury area (Supplementary Fig. 12c).

### Genetic ablation of *col12a1a*-expressing cells using the NTR/MTZ system

To investigate the role of *col12a1a*-expressing fibroblasts in heart regeneration, we used the fish line *Tg(-4kbcol12a1:GAL4VP16; UAS:NTR:RFP)*. The functionality of the cell ablation approach was verified in 48 h.p.f. embryos by treatment with a 5 mM solution of MTZ (Sigma-Aldrich) dissolved in DMSO that led to formation of RFP^+^ aggregates (Supplementary Fig. 12d), indicative of cell death as previously reported^37^. To ablate *col12a1a*-expressing cells in adult hearts, the injured fish were immersed 2 d.p.i. in a system of water containing 10 mM MTZ (or 0.2% DMSO as a vehicle control) and kept in the dark. Water with the fresh drug was exchanged twice a day until the day fish were euthanized. Fish were treated with MTZ from day 2 until day 6 for the 7 d.p.i. time point and also from day 14 to day 16 for the 30 d.p.i. time point. We observed increased mortality in col12a1a>NTR:RFP-ablated injured fish compared with DMSO-treated controls. Additional experiments would be needed to determine whether this mortality is related to heart regeneration defects.

### Cre–*lox* lineage tracing

For epicardial Cre–*lox* lineage tracing, *TgBAC(cryaa:EGFP, tcf21:Cre-ERT2; -3.5ubi-loxP-EGFP-loxP-mCherry)*-transgenic fish were treated with 10 μM 4-OHT (Sigma-Aldrich) dissolved in 100 ml of water per fish, 4 and 3 days before cryoinjury for 12 h as described^17^. Before systemic administration, the 10 mM stock (dissolved in ethanol) was heated for 10 min at 65 °C. Hearts were harvested 7 d.p.i. For endocardial Cre–*lox* lineage tracing, *Tg(fli1:Cre-ERT2; -1.5hsp70l:loxP-STOP-loxP-EGFP,cryaa:Venus)*-transgenic fish were treated as embryos at 24 h.p.f. with 5 μM 4-OHT (Sigma-Aldrich) for 24 h and heat shocked for 1 h at 37 °C. Adult fish were injured and hearts were harvested at 7 d.p.i.

### Statistics and reproducibility

Data collection and analysis were not performed blind to the conditions of the experiments. Sample collection and genomics analyses were performed by two independent experimenters. Sample sizes (*n*) are indicated either in each figure legend including the number of independent experiments or in this section. Quantification of each experiment is described in the respective section of the Methods together with the software used. No data points were excluded from the analysis. Graphs and statistical analyses shown in Figs. 3h,j and 6b,g,i and in Supplementary Figs. 12f and 24b were performed in GraphPad Prism (v.7 and v.9). Graphs show mean and s.d.; the statistical tests with *P* values are indicated in figure legends. The experiments in Fig. 3g used DMSO-treated fish (*n* = 3) and MTZ-treated fish (*n* = 6) at 7 d.p.i; *n* represents biologically independent samples from two independent experiments. The experiments in Fig. 3i used DMSO-treated (*n* = 4) and MTZ-treated (*n* = 3) fish; results are from two independent experiments. All RNAscope experiments in Figs. 3b and 6c,e and in Supplementary Figs. 7, 8 and 12 were performed at least three times using one or two independent experiments, unless stated otherwise. Fig. 4e (*n* = 3) shows the results of one independent experiment, Fig. 5e (*n* = 2) shows the results of one independent experiment; for the experiments in Fig. 6c,d, *n* = 9, 3, 9, 9 animals; Fig. 6b, lower right panel: DMSO was injected at 3 d.p.i., *n* = 6; 7 d.p.i., *n* = 9, 15 d.p.i., *n* = 3, and 30 d.p.i., *n* = 8, and IWR-1 was injected at 3 d.p.i., *n* = 5; 7 d.p.i., *n* = 9; 15 d.p.i., *n* = 3, and 30 d.p.i., *n* = 8; *n* represents biologically independent samples over four independent experiments. In Fig. 6g, *n* represents biologically independent samples over two independent experiments; in Fig. 6i, *n* represents biologically independent samples over two independent experiments. The results in Supplementary Fig. 3d are based on *n* = 2 animals in one independent experiment, those in Supplementary Fig. 24c (*n*_5s_ = 3, *n*_10s_ = 3, *n*_15s_ = 3, *n*_20s_ = 4, *n*_25s_ = 3) are based on two independent experiments. The number of animals was determined based on previous literature^6,10,17,41,68,69^ for the experiments in Figs. 3b, 4e, 5e and 6f–I and in Supplementary Figs. 3d, 7, 8 and 12b,c or calculated using G*power calculator^70^ by two-tailed *t* test with α err prob = 0.05 and power(1-β err prob) = 0.80; effect sizes *f* were determined as follows: Fig. 3g–j and Supplementary Fig. 12e,f: *f* = 2; Fig. 6b,c,e: *f* = 1.5; Supplementary Fig. 24c: *f* = 1.48.

### Mapping and filtering of single-cell scar data

Scar filtering was performed as described by Spanjaard et al.^24^. Briefly, scar sequences were aligned using *bwa mem*^3^ (v.0.7.12) to a reference of dTomato. Valid cell barcodes were identified based on the single-cell transcriptome data (see previous paragraph). We removed reads that were unmapped, had an incorrect barcode or did not start with the exact PCR primer we used. We truncated all scar sequences to 75 nucleotides and removed shorter sequences.

In further filtering steps, we consecutively filtered out scars in order to remove all scars that did not fulfill the following quality control conditions. As a first step, we required all molecules to be sequenced at least twice to remove straightforward sequencing errors; this also reduced complexity for the subsequent filtering steps. To remove molecules caused by incorrect annealing, we selected only the most prevalent scar for each combination of barcode and unique molecular identifier (UMI), only the most prevalent UMI for each barcode and scar, and only the most prevalent barcode for each UMI and scar. We then assessed similar (Hamming distance of 2 or less) scar sequences in each cell, only keeping both if they met criteria designed to test whether both sequences were verifiably correct (see Spanjaard et al.^24^ for details). Finally, we removed cells that looked like doublets by comparing their numbers of different scars with the scar number distribution for that cell type.

### Tree building

Tree building was done as described in Spanjaard et al.^24^. Briefly, the lineage tree building algorithm was designed to build lineage trees with high numbers of cells, despite the low detection rate inherent in single-cell sequencing. It consisted of two steps: we first determined the lineage tree in terms of scars created and afterwards placed the single cells in their appropriate positions in the tree.

To build the scar lineage, we used the data set of scars in single cells and determined which scars could be found in the same branch of the lineage trees: those scars were observed together in the same cell. We created a graph of scars with connections between scars, indicating that they were together in a branch. A scar that had been created early would be present in many branches and connected to many other scars. As a corollary, the scar that was connected to most other scars in this graph was created before all other scars. This scar became the root of the lineage tree; it was removed from the scar graph and we identified the most highly connected scar in the resulting graph. This scar was placed underneath the first scar in the lineage tree and was again removed from the graph. If at any point the graph split into disconnected parts after removing a scar, those parts were considered to be distinct branches of the lineage tree and were treated separately. This process was repeated until all scars had been placed in the lineage tree.

The above tree construction algorithm is valid for high, nonvariable scar-detection rates with perfect filtering and no doublets; for more realistic scenarios, computations are more complicated but follow the general scheme as outlined above. Refer to Spanjaard et al. for details^24^.

Once the shape of the lineage tree and the location of all scars in the lineage tree had been determined, we placed the cells in the tree. Cells were placed using the scars that were found in them, at the lowest possible position in the tree. Importantly, this means that some cells may have been placed in leaf nodes, but typically some cells in which a leaf node scar had not been detected would be placed in internal nodes.

### Lineage determination

To determine lineage relationships between cell types, we contracted all vertical branches (that is, removed all nodes that were not part of a lineage split) and then calculated cell type ratios – the amount of cells of a specific type divided by the node size – for each node. We calculated the lineage similarity between two cell types as the correlation between their cell type ratios, weighted by the size of the nodes. Importantly, this approach allowed us to integrate lineage data for multiple biological replicates. Using cell type ratios emphasizes nodes that are lower in the trees and contain fewer cell types, and weighting by node size emphasizes larger nodes to maximize reproducibility.

We determined the stability of clusters in two different ways. A thousand-fold repetition of downsampling all lineage trees to half of the cells was used to estimate the stability of lineage clusters against low cell numbers. After calculating the correlations and clustering the cell types, we recovered the same clusters in the majority of cases, indicating that these clusters were not influenced by low cell numbers (Supplementary Fig. 19). A thousand-fold repetition of downsampling all lineage trees to half of the nodes was used to estimate the stability of lineage clusters against low clonality or clone-specific transition rates. After calculating the correlations and clustering the cell types, we found that *nppc*, *spock3*, *cfd* and valve fibroblasts were attributed to varying clusters, indicating that differences between clones were too large for the correlation analysis to yield reliable answers (Supplementary Fig. 22).

To determine possible sources of *nppc*, *spock3, cfd* and valve fibroblasts, we determined the overlap of cell types with these target cell types in tree leaf nodes. We calculated the conditional probability of cell types as the number of leaf nodes that contained cells of both source and target type divided by the number of leaf nodes that contained cells of the target type. For example, at 7 d.p.i., ventricular endocardium had a conditional probability of 1 with regard to *nppc* fibroblasts, meaning that at 7 d.p.i., every leaf node that contained *nppc* fibroblasts also contained ventricular endocardium. However, *nppc* fibroblasts had a conditional probability of 0.2 with regard to the ventricular endocardium, indicating an asymmetric lineage relationship between these cell types.

### Trajectory analysis and RNA velocity

We used PAGA, implemented in scanpy^38^ (v.1.8.2) and scvelo^71^ (v.0.2.4) for integrated trajectory and RNA velocity analysis. First, we extracted velocity information from reads aligned to a transcriptome based on GRCz11, release 92, using Cell Ranger 3.1 and velocyto^39^. Using cell type annotations determined previously, we selected relevant cells; regressed out total counts, numbers of genes and mitochondrial percentages; determined highly variable genes and principal components; and used bbknn to integrate data sets. We then reclustered the data using the Leiden algorithm and ran PAGA on the Leiden clusters. For the integrated data set, we calculated RNA velocities using the stochastic model implemented in scvelo and plotted those on the UMAP representation of the data.

### Secretome analysis

We performed an ortholog conversion of the Vertebrate Secretome Database^72^ using orthology data from the Alliance of Genome Resources, release 3.2.0 (ref. ^73^). For the ortholog conversion, we used all gene pairs with ‘IsBestScore=Yes’ and kept all unique zebrafish gene names. After normalizing all cells to 10,000 transcripts, we calculated the percentage of transcripts that were in the transcriptome for each cell type at each time point. We then calculated log(1 + x) of the average expression of secretome genes per cell type and used the clustermap function in seaborn to perform hierarchical clustering using Ward’s method for the cell types and secretome genes that were expressed at an average of 10 in at least one cell type. We similarly performed hierarchical clustering of cell type averages of *z* transformed secretome expressions (capped at 5) for genes that had a *z* score greater than 2 in at least one cell type. We excluded neuronal cells and myelin cells from these analyses.

### Interaction analysis

We used CellPhoneDB^74^ (v.2.1.4) to analyse interactions between cell types at each time point after ortholog conversion of the genes in CellPhoneDB, similar to the conversion of the secretome. We included all cell types with more than 50 cells or whose cell count proportion of the total data set exceeded 0.01 and counted the number of significant outgoing and incoming interactions for each cell type.

### tomo-seq data deconvolution

We used AutoGeneS^29^ (v.1.0, https://github.com/theislab/AutoGeneS) to deconvolve the tomo-seq data. As input, we used the single-cell data constrained to the 5,000 most variable genes using scanpy^75^ (pp.highly_variable_genes). AutoGeneS was then used to select a total of 400 informative genes from among the highly variable ones that differentiated the cell types. The highly variable genes were selected based on normalized dispersion. The proportions were inferred using nonnegative least squares^76^ (scipy.optimize.nnls) based on the cellular mean expression of the informative genes. For each bulk sample, negative proportions were set to zero and the rest were normalized to sum to one.

### Reporting summary

Further information on research design is available in the Nature Research Reporting Summary linked to this article.

## Online content

Any methods, additional references, Nature Research reporting summaries, source data, extended data, supplementary information, acknowledgements, peer review information; details of author contributions and competing interests; and statements of data and code availability are available at 10.1038/s41588-022-01129-5.

## Supplementary information

Supplementary Information Supplementary Figs. 1–26, Table 1 and statistical source data for Figs. 1–26.

Reporting Summary

Supplementary Data 1 Sequencing library statistics.

Supplementary Data 2 Marker genes for the clusters identified in Fig. 1b.

Supplementary Data 3 Marker genes for subclusters.

Supplementary Data 4 Cell type counts in the individual sequencing libraries.

Supplementary Data 5 Enrichment of secreted genes in all cell types (*z* scores).

## Data Availability

Source data are provided with this paper. Sequencing data have been deposited in the Gene Expression Omnibus with accession numbers GSE159032 and GSE158919. Transcriptome data were aligned to a zebrafish transcriptome created with Cell Ranger 3.0.2 from GRCz11, release 92. We performed an ortholog conversion of the Vertebrate Secretome Database VerSeDa using orthology data from the Alliance of Genome Resources, release 3.2.0.

## Source data

Source Data Fig. 1 Statistical source data for Fig. 1.
Source Data Fig. 2 Statistical source data for Fig. 2.
Source Data Fig. 3 Statistical source data for Fig. 3.
Source Data Fig. 4 Statistical source data for Fig. 4.
Source Data Fig. 5 Statistical source data for Fig. 5.
Source Data Fig. 6 Statistical source data for Fig. 6.

## Code availability

Custom code is provided at https://github.com/Bastiaanspanjaard/Heart_regeneration.
