# Supplementary material for: Origin and function of activated fibroblast states during zebrafish heart regeneration
Source: Nat Genet. 2022 Jul 21;54(8):1227–37. doi: 10.1038/s41588-022-01129-5 (PMC7613248; doi:10.1038/s41588-022-01129-5)
Supplement: Supplementary file 1 — Supplementary Figs. 1–26, Table 1 and statistical source data for Figs. 1–26. [file 41588_2022_1129_MOESM1_ESM.pdf]

nature portfolio

https://doi.org/10.1038/s41588-022-01129-5

Supplementary information

Origin and function of activated fibroblast states during zebrafish heart regeneration

In the format provided by the authors and unedited

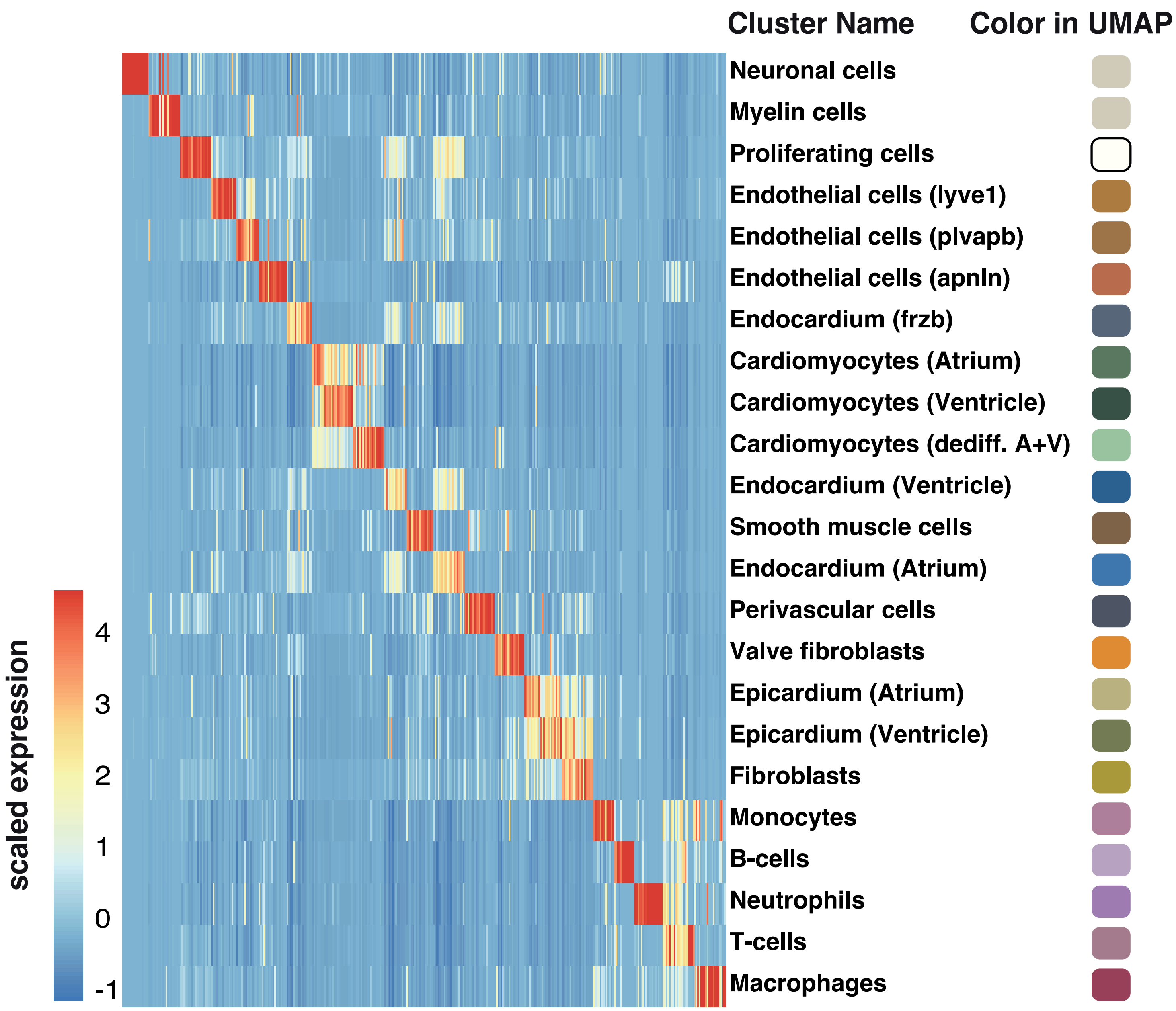

Figure S1. Top differentially expressed genes for each cell type shown in Fig. 1b. The genes used in this heatmap can be found in Table S2. The legend on the right shows the color of the respective cell type in Fig. 1b.

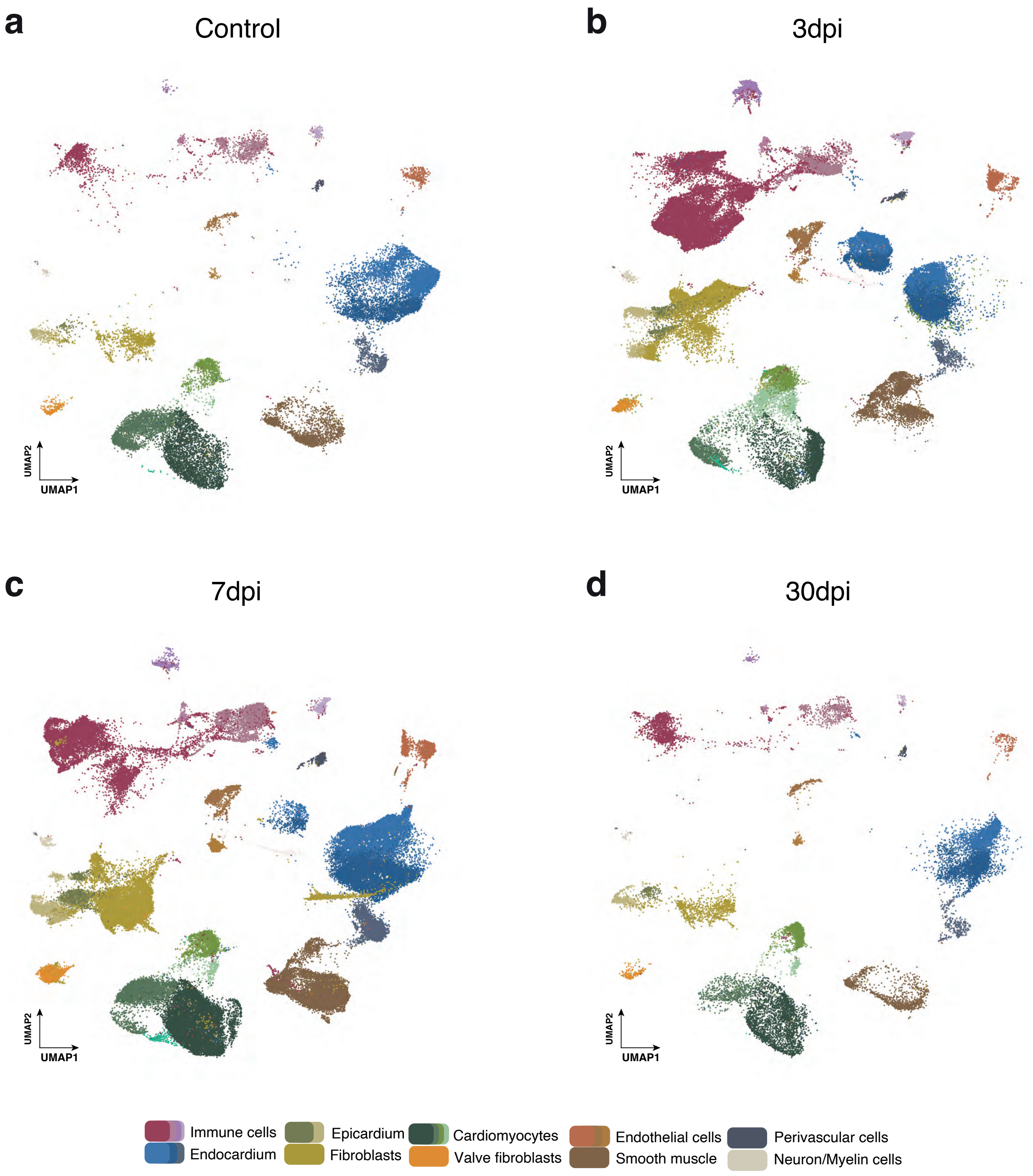

Figure S2. Single-cell datasets at control (a), 3 dpi (b), 7 dpi (c) and 30 dpi (d).

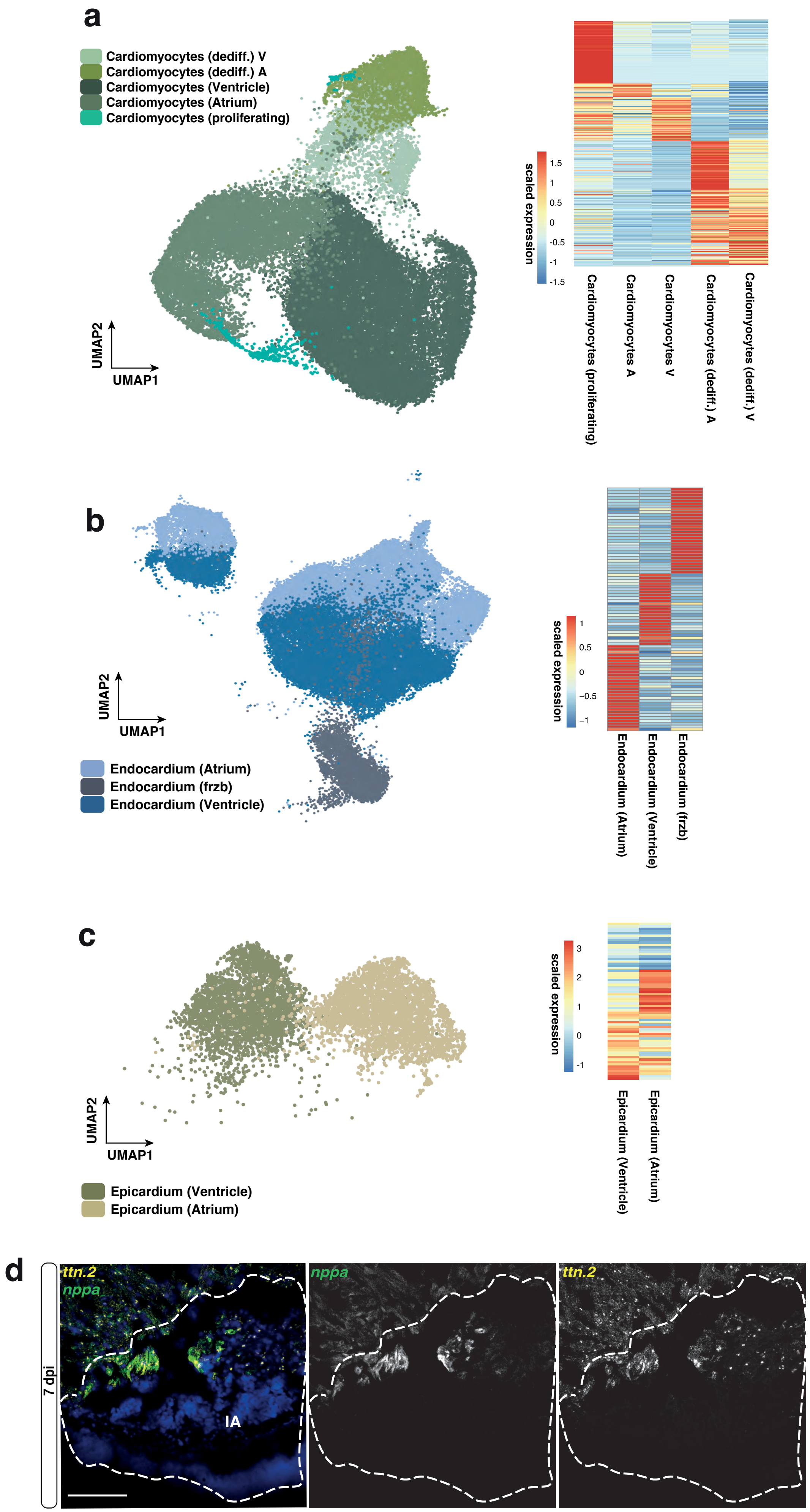

Figure S3. Subclustering of cardiomyocytes (a), endocardium (b), and epicardium (c). Heatmaps show top differentially expressed genes of each subtype. The genes used for the heatmap can be found in Table S2. The UMAP representation of the clustering is zoomed in from Fig. 1B. **(d)** Dedifferentiated CMs labeled with *ttn.2* and *nppa* as markers at 7 dpi. White dashed lines indicate the injury area (AI). Scale bar: 100μm.

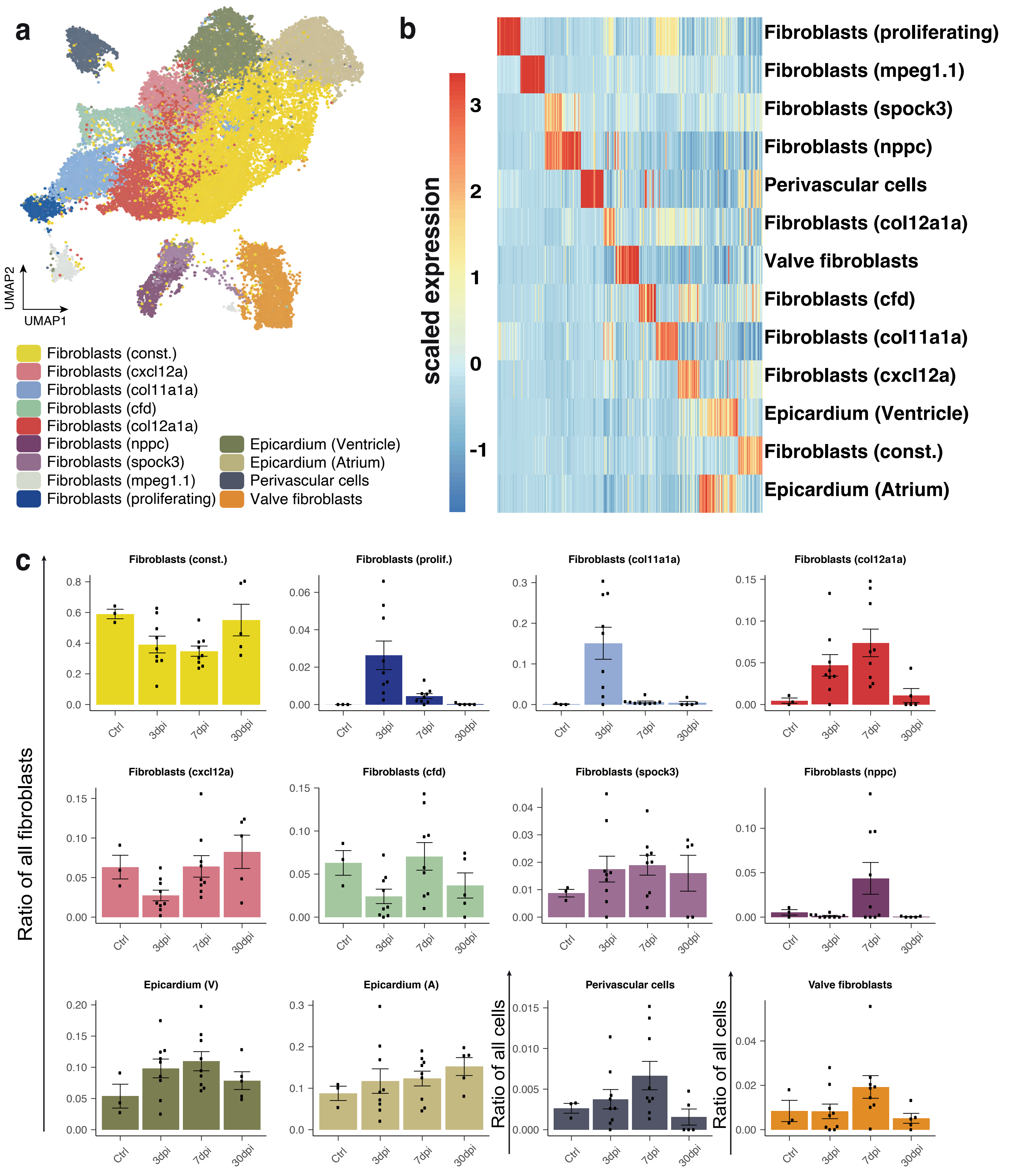

Figure S4. Subclustering of collagen expressing cells. (a) UMAP representation of subclustering results. Our analysis identified 13 subclusters. **(b)** Top differentially expressed genes in each subcluster. Gene names can be found in Table S3. **(c)** Cell number dynamics of all subtypes during regeneration across the timepoints (n = 3, 9, 9, 5 animals; error bars show standard error of the mean).

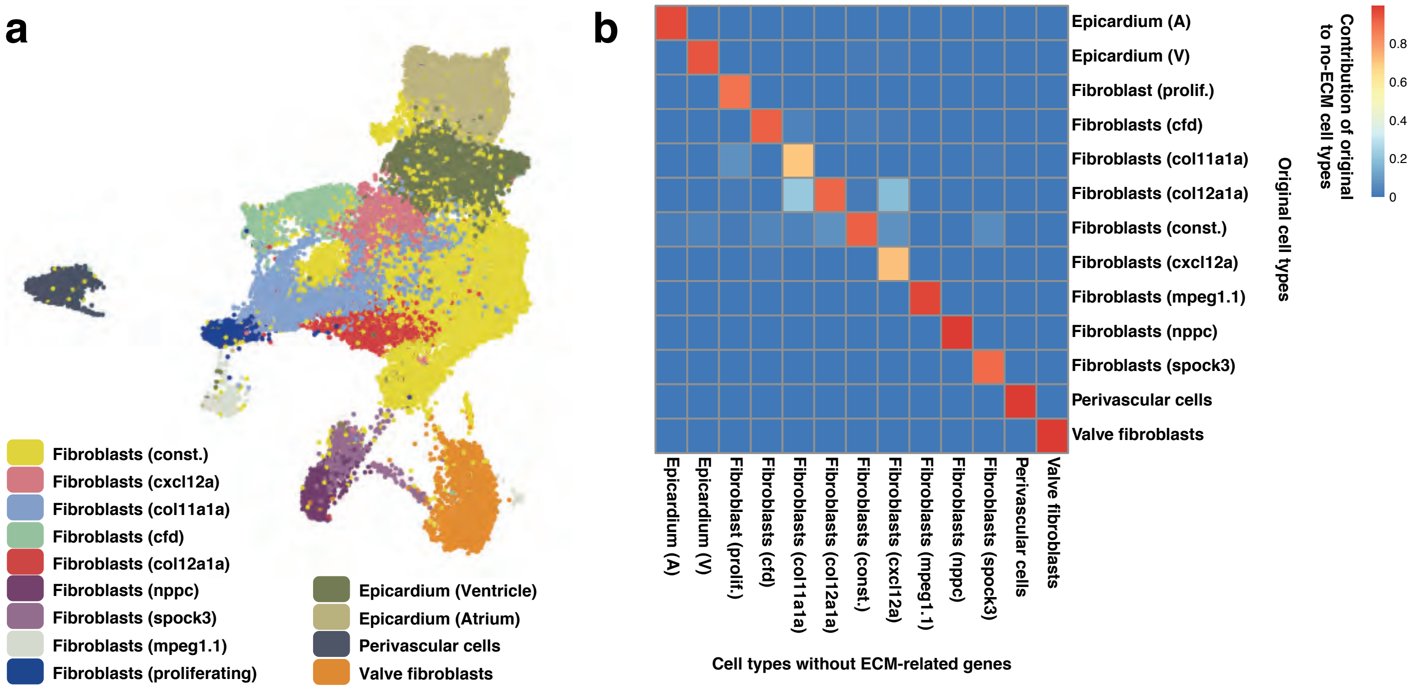

Figure S5. Fibroblast subtypes are distinct without ECM-related genes. (a) UMAP and clustering is left largely invariant after removal of ECM-related genes. **(b)** Cell types identified after removal of ECM-related genes largely overlap with original cell types, although a small fraction of *col12a1a* fibroblasts is found in the *col11a1a* and *cxcl12a* fibroblast subtypes.

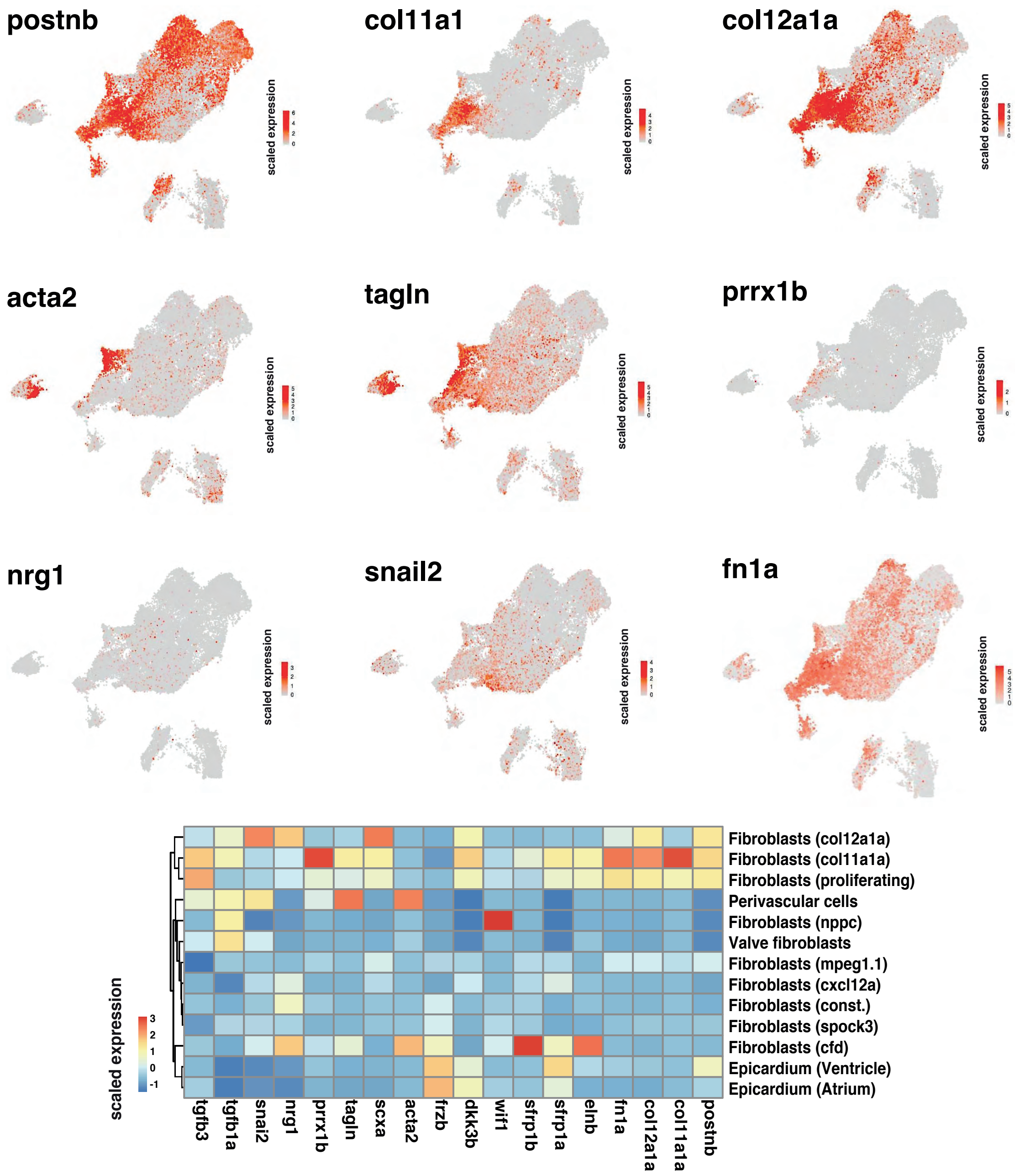

Figure S6. Gene expression in fibroblast subtypes. Expression of selected genes involved in fibroblast function or heart regeneration in the identified fibroblast subclusters.

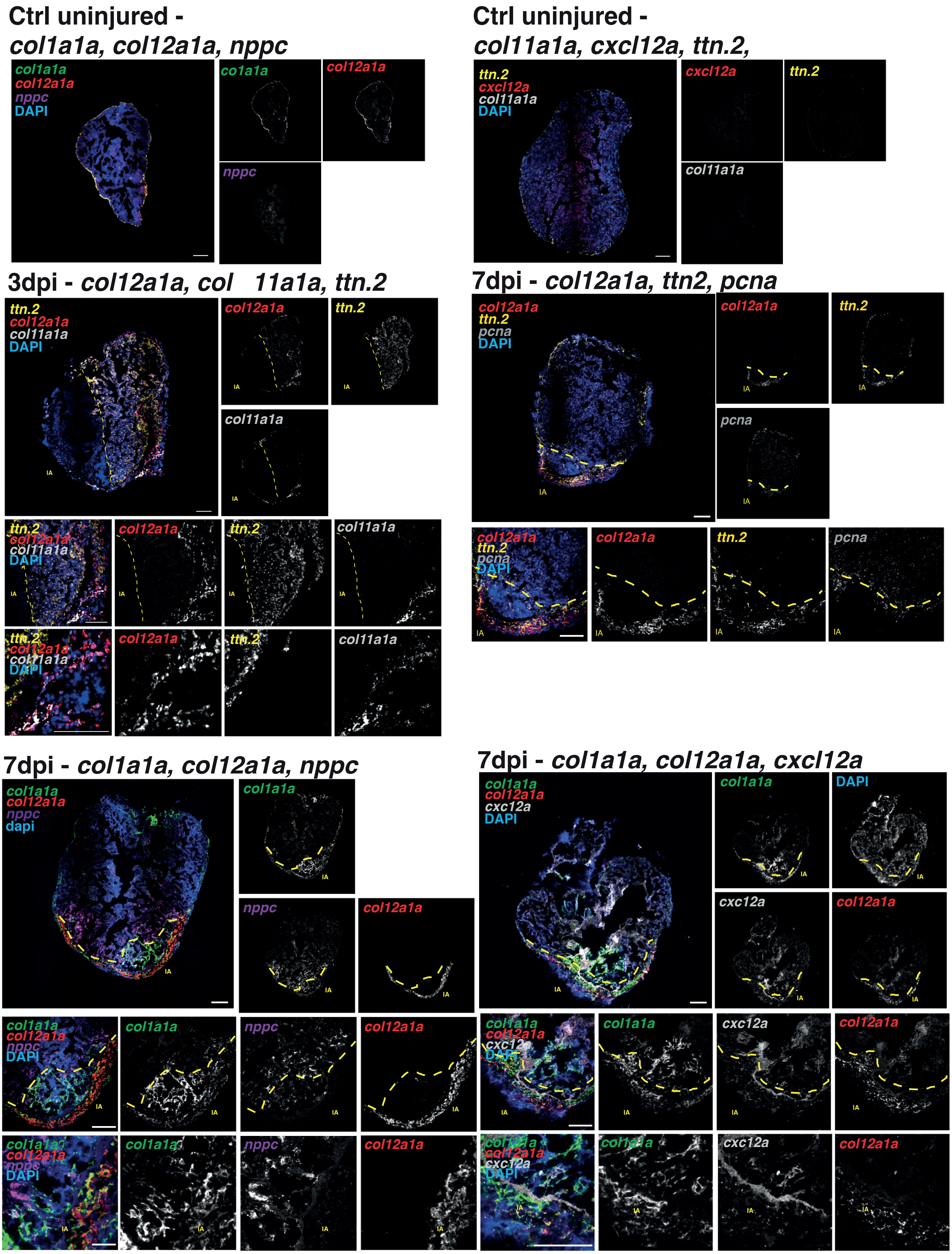

Figure S7. Fluorescent *in situ* hybridization of marker genes for cell types of interest at different time points of injury. Markers for transient cell types (*col12a1a*, *col11a1a*, *ttn.2* for dedifferentiated cardiomyocytes, *nppc*) are localizaed at the injury area. Yellow dashed lines indicate injury area (IA). Scale bar: 100 μm.

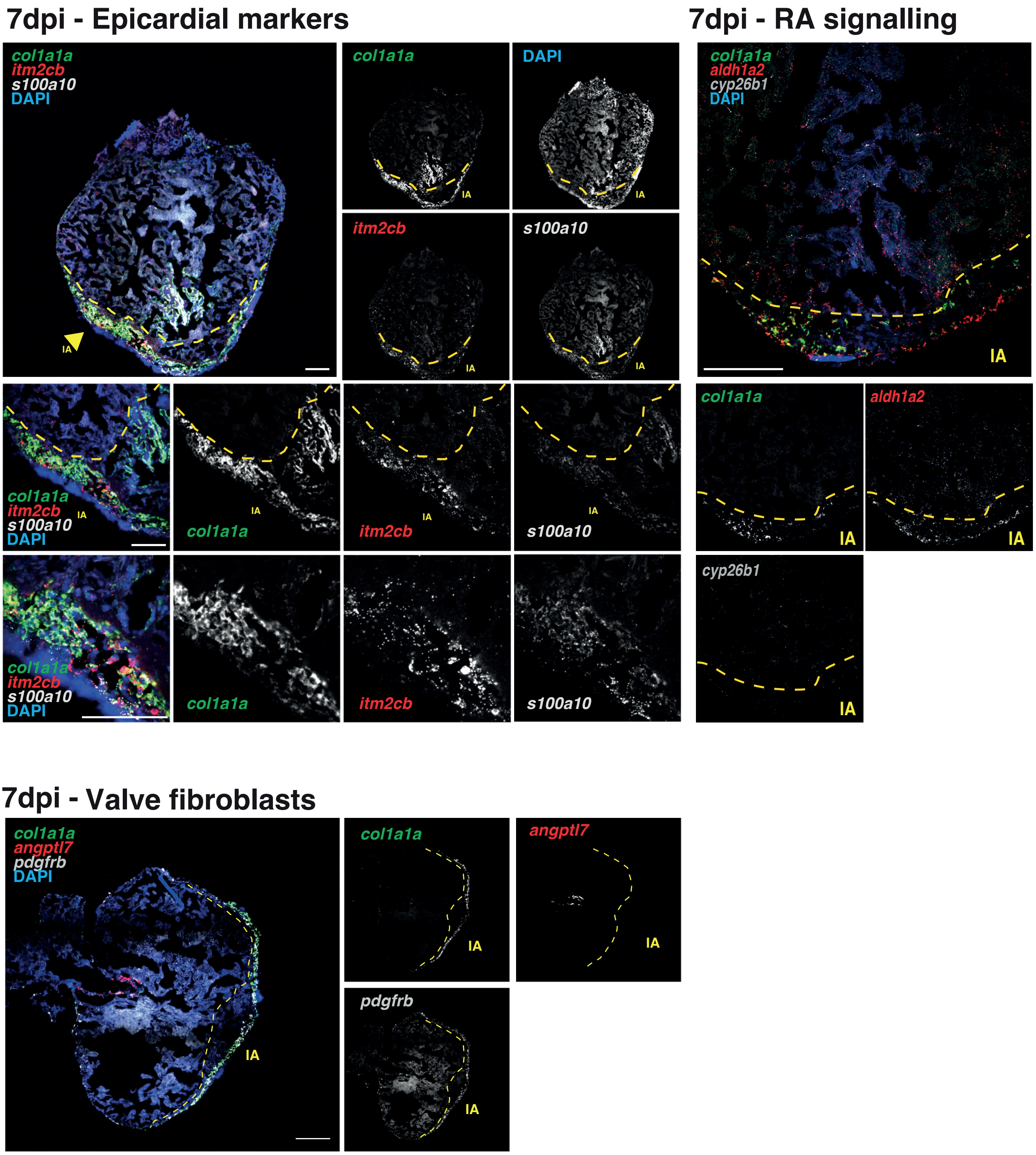

Figure S8. Fluorescent *in situ* hybridization of marker genes for additional cell types of interest and factors of the Retinoic acid (RA) signalling pathway. Epicardium - *itm2cb*, *s100a10*; valve fibroblasts - *angptl7*; perivascular cells - *pdgfrb*; RA signaling - *aldh1a2, cyp26b1*. Yellow dashed lines indicate injury area (IA). Scale bar: 100 μm.

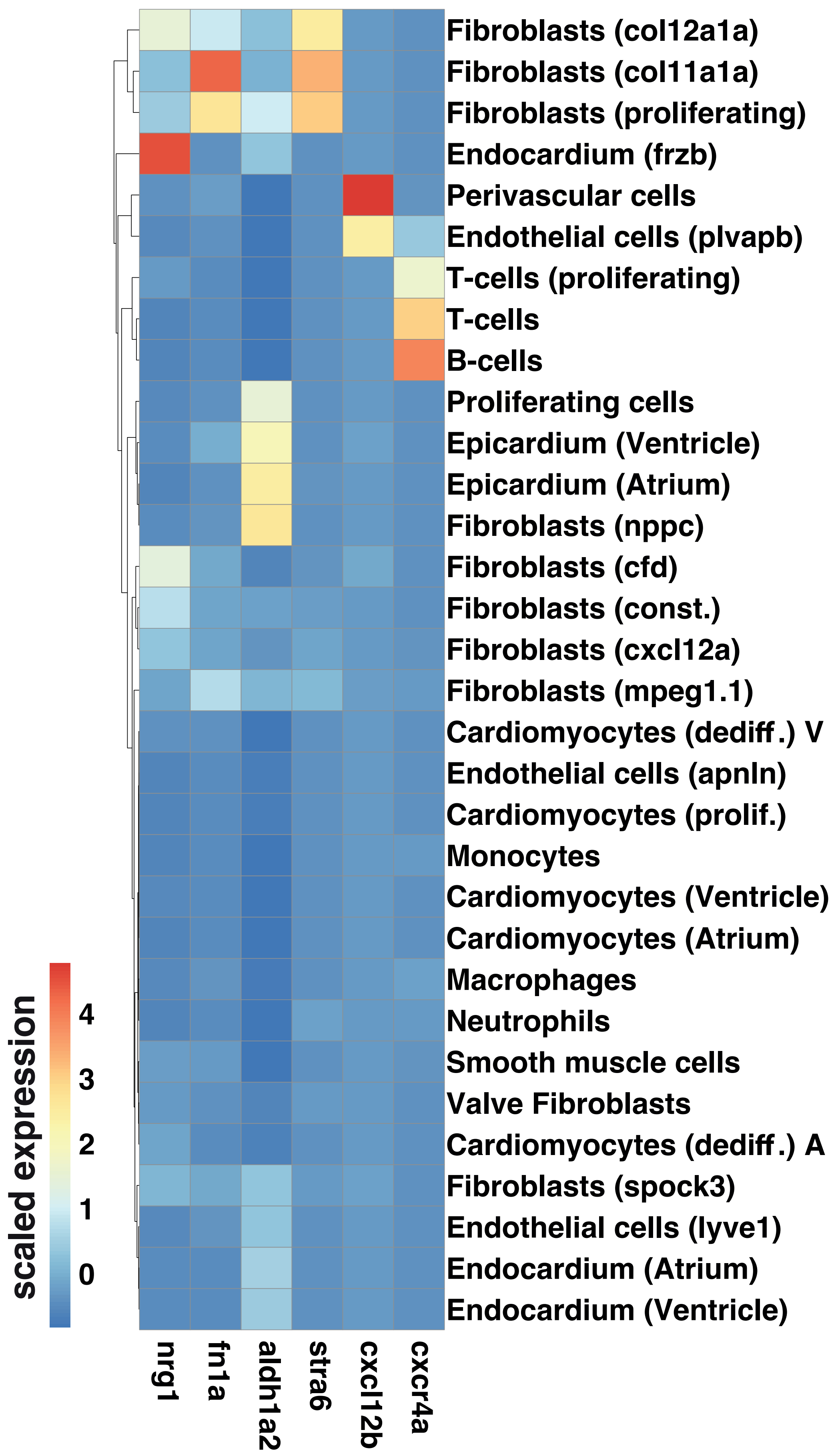

Figure S9. Expression of known pro-regenerative factors across additional cell types of the zebrafish heart. Expression patterns of *cxcl12b*, *cxcr4a*, *adlh1a2*, *stra6*, *nrg1* and *fn1a*. See main text for interpretation of the expression patterns.

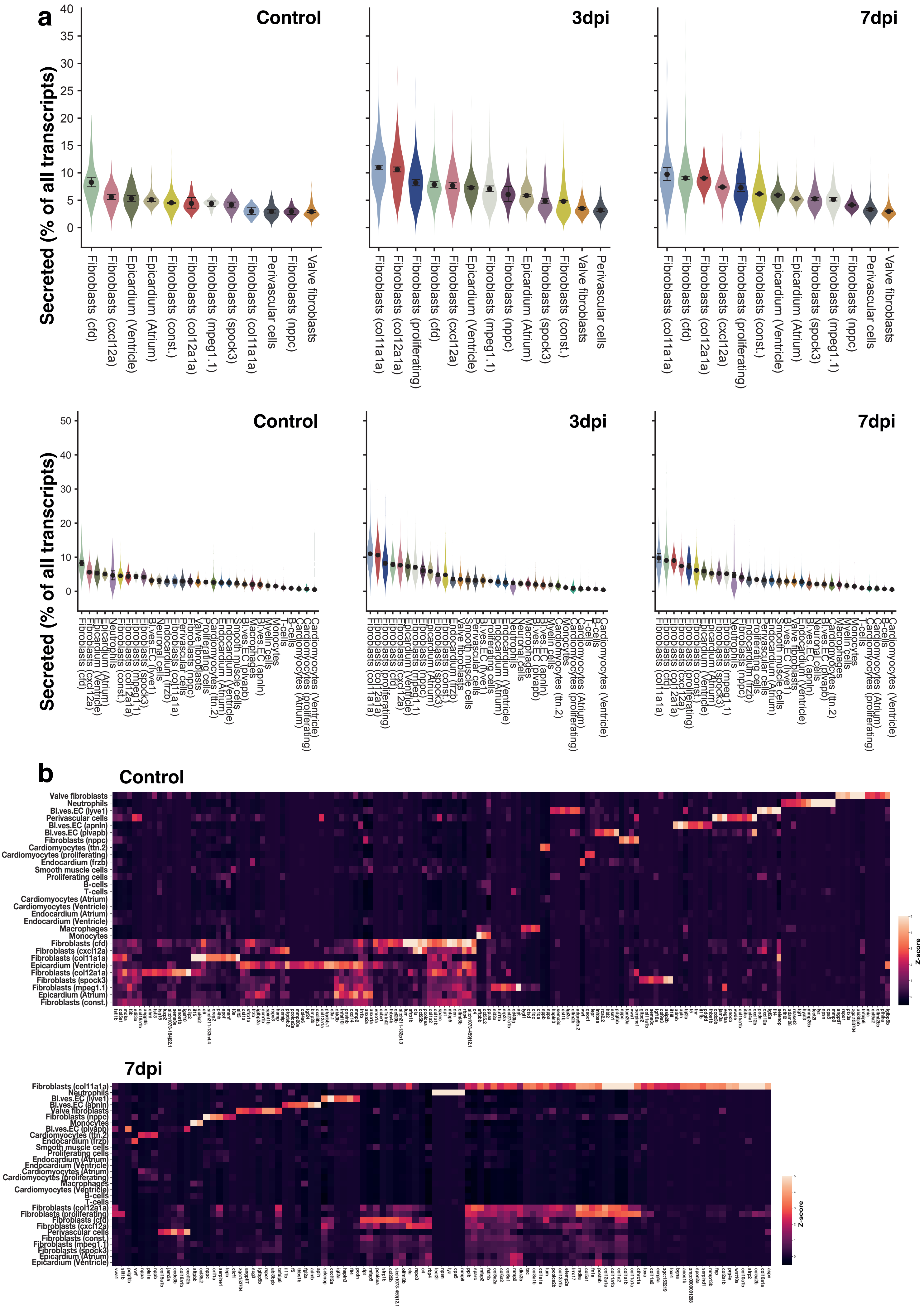

Figure S10. Secretome analysis. (a) Mean secretome expression of fibroblast (n=1947,8611,17261 cells) and all cell types (n=23010,54834,82215 cells). Error bars are 3*standard error of mean. **(b)** Highly expressed secretome genes in control hearts and at 7 dpi.

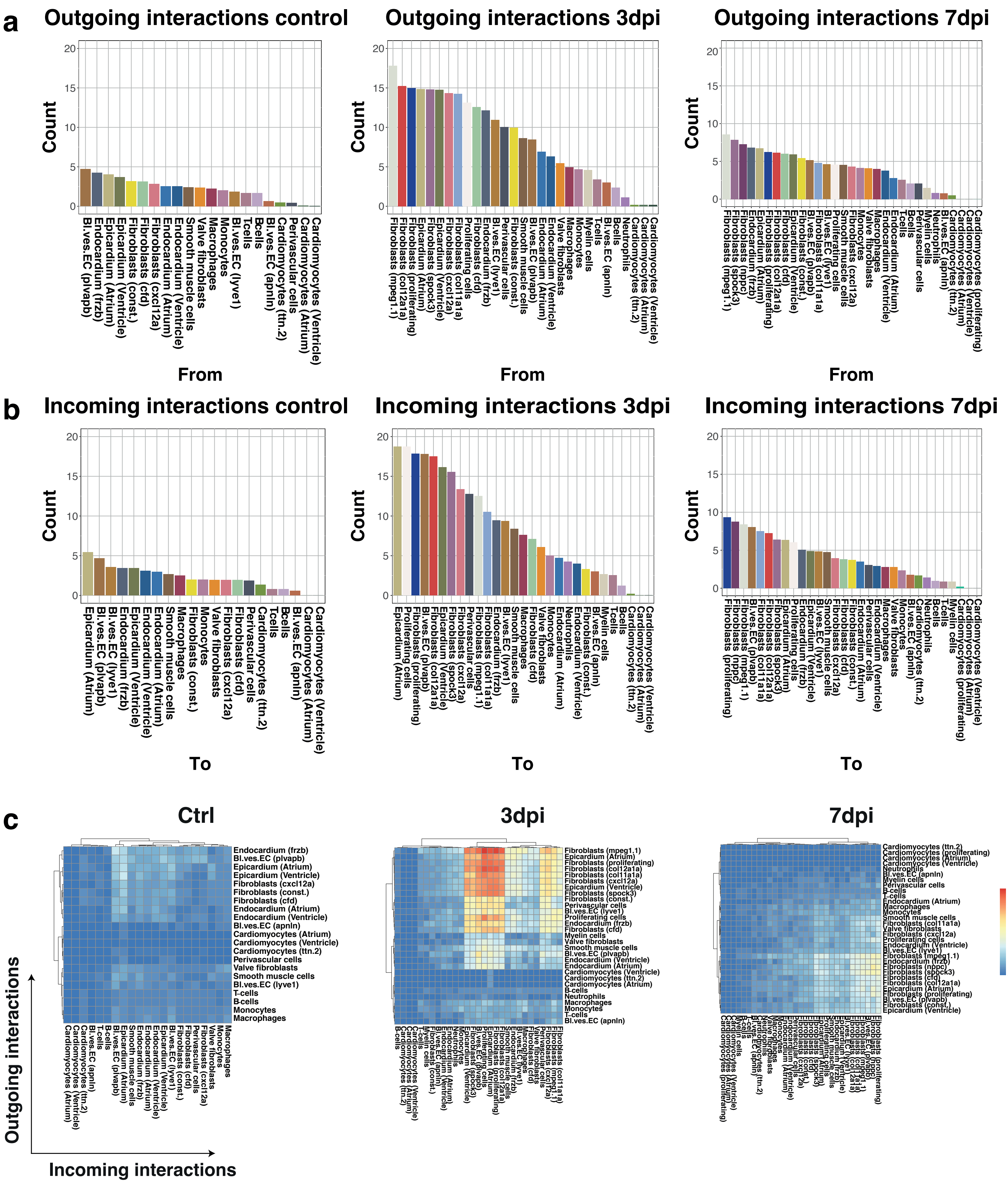

Figure S11. Ligand-receptor analysis for cell-cell interactions. (a) Number of outgoing interactions (ligands) per cell type. **(b)** Number of incoming interactions (receptors) per cell type. **(c)** Interactions between pairs of cell types.

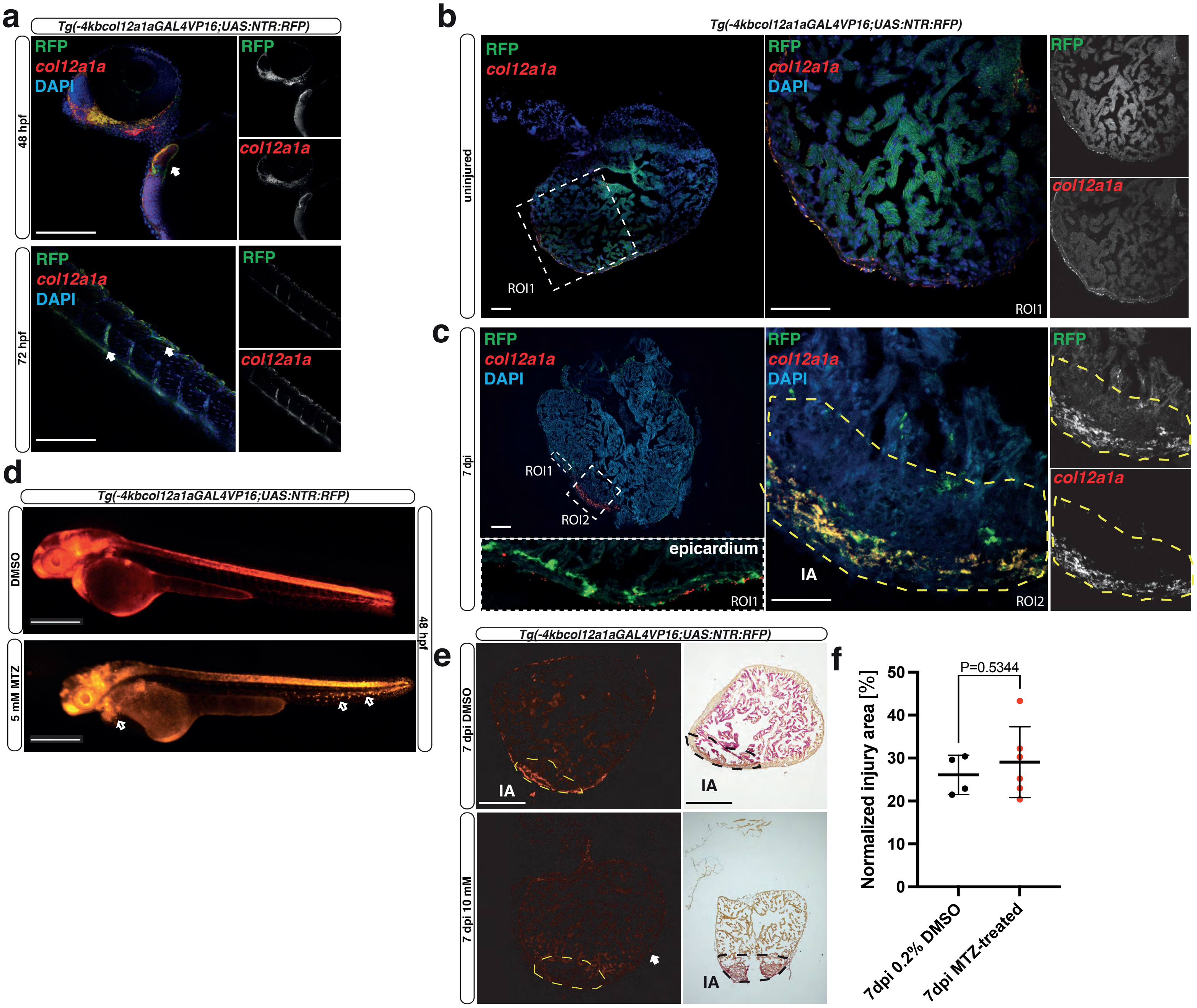

Figure S12. Characterization of *Tg(-4kbcol12a1aGAL4VP16;UAS:NTR:RFP)* line. (a) Embryos at 48 hpf and 72 hpf showing expression of *col12a1a* in developing embryo (arrows: heart, somites) co-localizing with RFP signal. Scale bar: 100 μm. **(b)** Uninjured adult heart showing the expression of *col12a1a* in the epicardium (*col12a1a* in red, RFP in green). Region of interest (ROI) in the left panel as white dashed rectangle is shown at higher magnification in the panels on the right. Scale bar: 100 μm. **(c)** Expression of *col12a1a* in the injury area and the epicardium at 7dpi co-localizes with RFP signal (*col12a1a* in red, RFP in green). Regions of interest (ROI 1 & 2) in the upper left panel (white dashed rectangles) are shown at higher magnification in the panels below and on the right. Yellow dashed lines indicate injury area (IA). Scale bar: 100 μm. **(d)** Fluorescent images of embryos at 48 hpf treated with 0.1% DMSO or 5 mM of MTZ. Arrows point to aggregates of *RFP* in *Tg(-4kbcol12a1aGAL4VP16;UAS:NTR:RFP)* embryo after treatment with MTZ. Scale bar: 500 μm. **(e)** Fluorescent images of 0.2% DMSO- and 10mM MTZ-treated hearts at 7dpi. Arrows point to *col12a1a* aggregates. Yellow dashed lines indicate injury area. Histological comparison of the injury area at 7 dpi with or without MTZ treatment. Scale bar: 300μm. **(f)** Relative size of the injury area across all histological replicates at 7 dpi in 0.2% DMSO- (n=4), and MTZ-treated (n=6) hearts, mean and standard deviation are shown. Student's t-test.

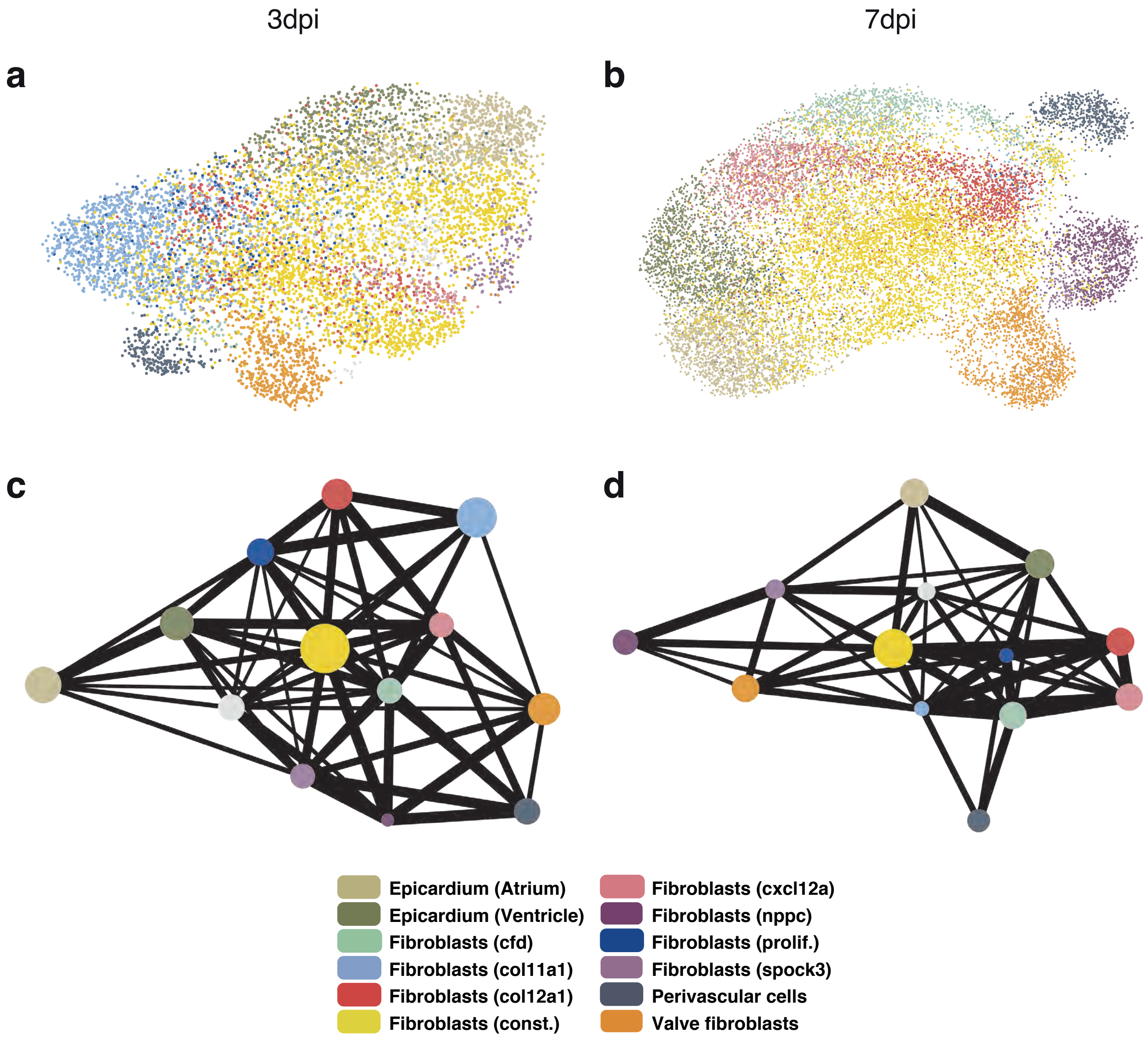

Figure S13. Trajectory analysis on mRNA identifies connections between all niche fibroblasts. (a-b) UMAP representation of cell types in the epicardial/fibroblast niche at 3 dpi **(a)** and 7 dpi **(b)**. **(c-d)** All cell types are connected with a strength of at least 0.3 in a PAGA analysis at 3 dpi **(c)** and 7 dpi **(d)**.

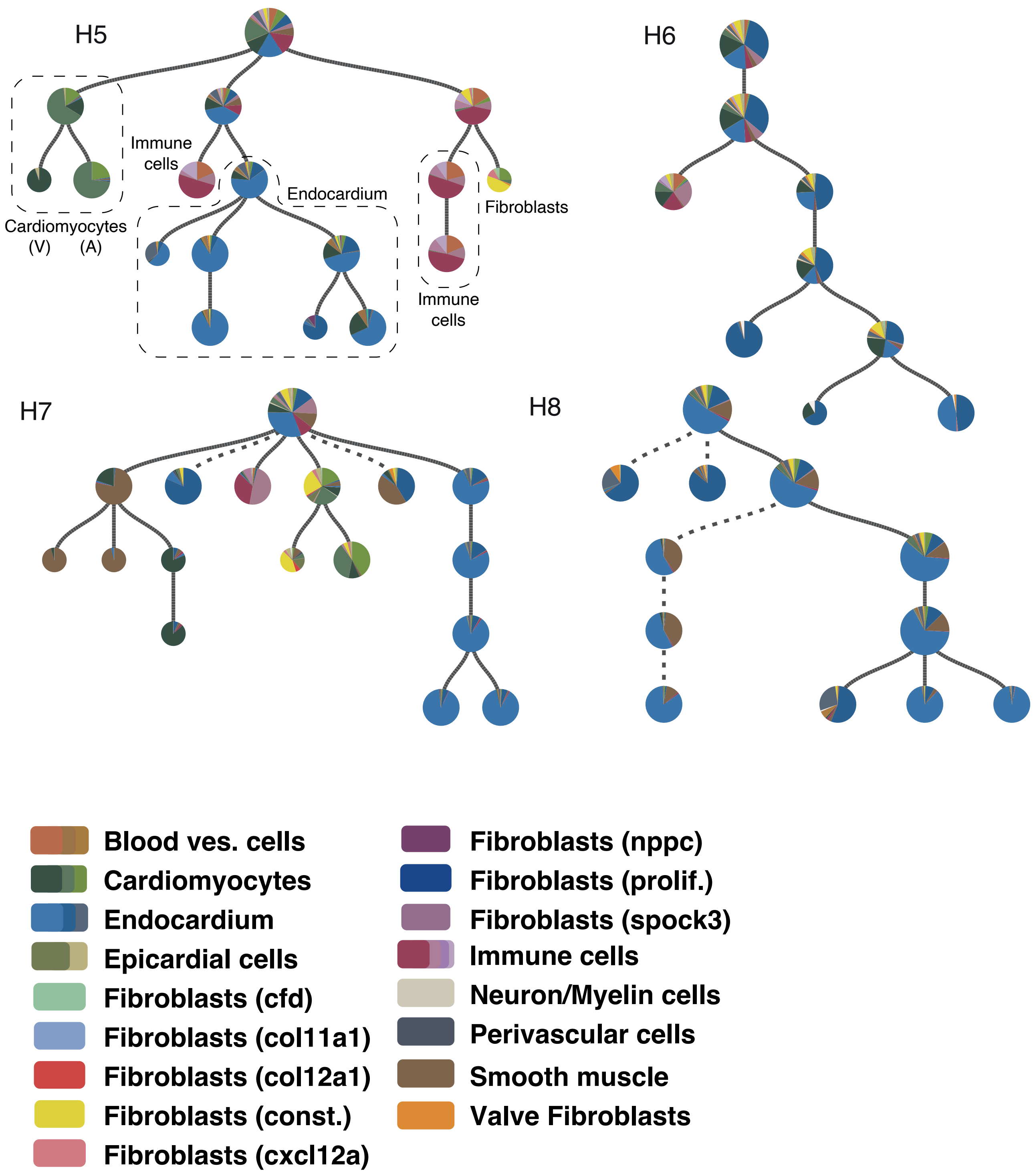

Figure S14. Lineage trees pre-injury. In H5, branches are annotated by majority cell type.

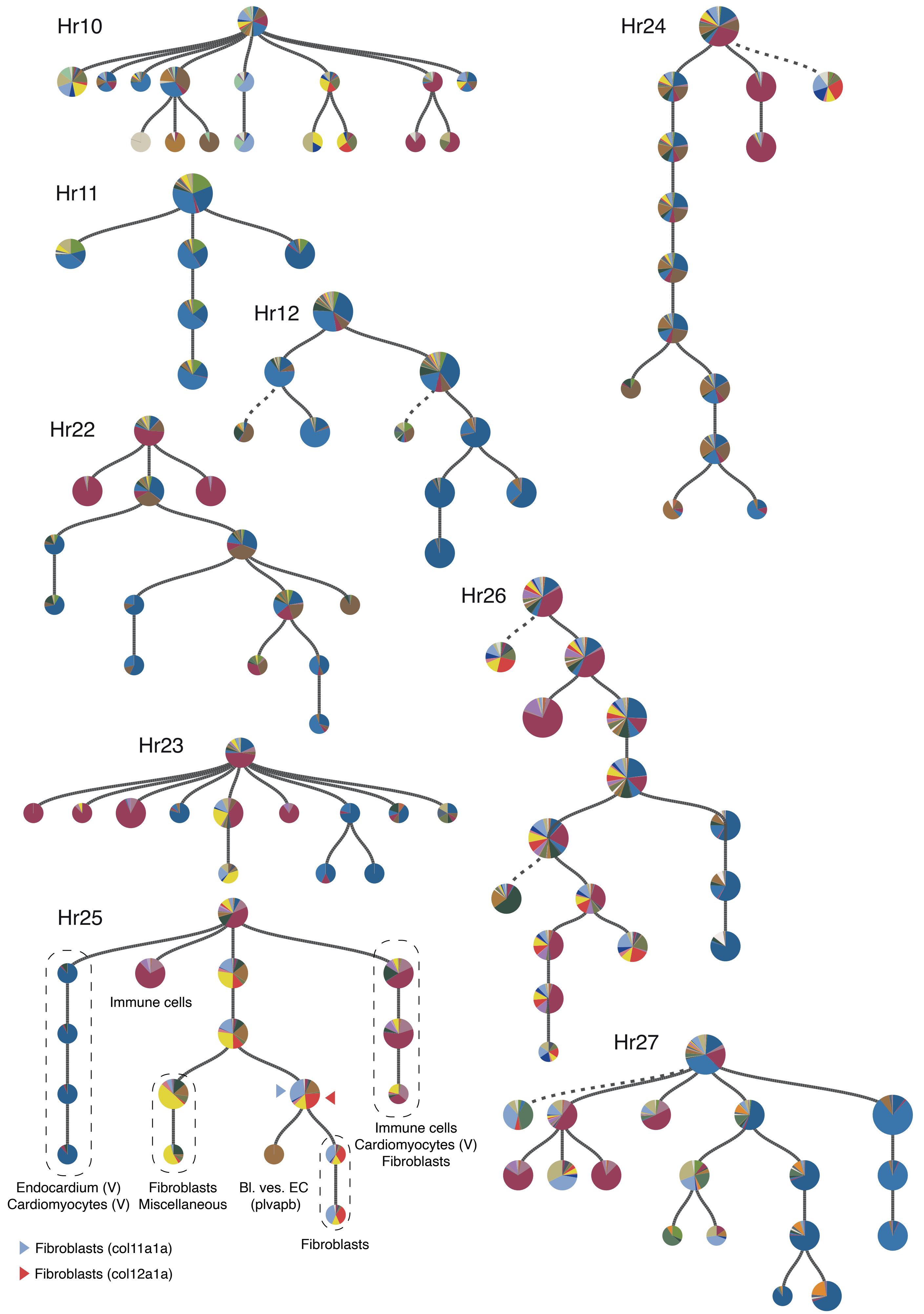

Figure S15. Lineage trees three days post injury. See Fig. S14 for cell type colors. In Hr25, branches are annotated by majority cell type; arrowheads indicate transient col11a1a and col12a1a fibroblasts.

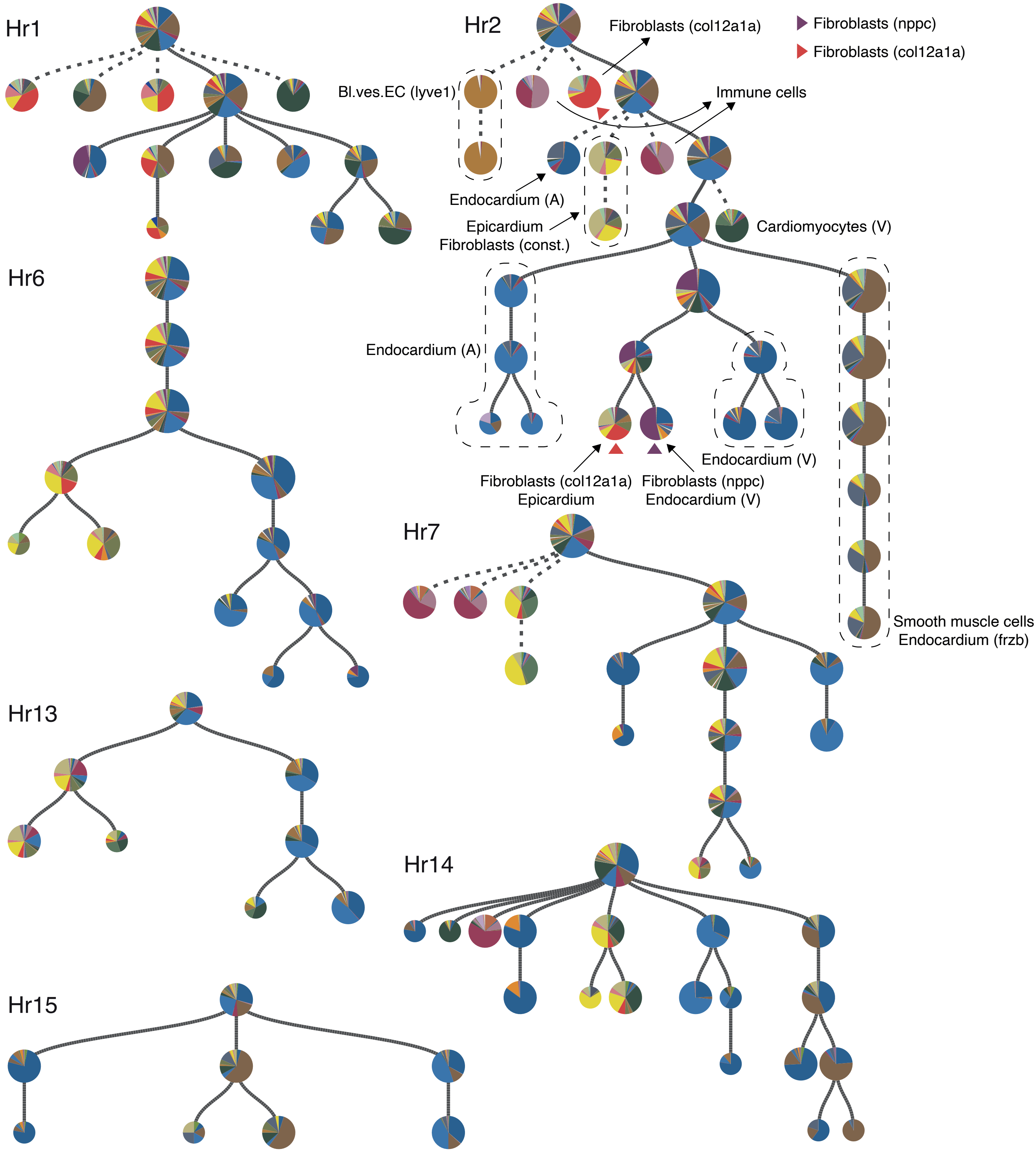

Figure S16. Lineage trees seven days post injury. See Fig. S14 for cell type colors. In Hr2, branches are annotated by majority cell type; arrowheads indicate transient col12a1a and nppc fibroblasts.

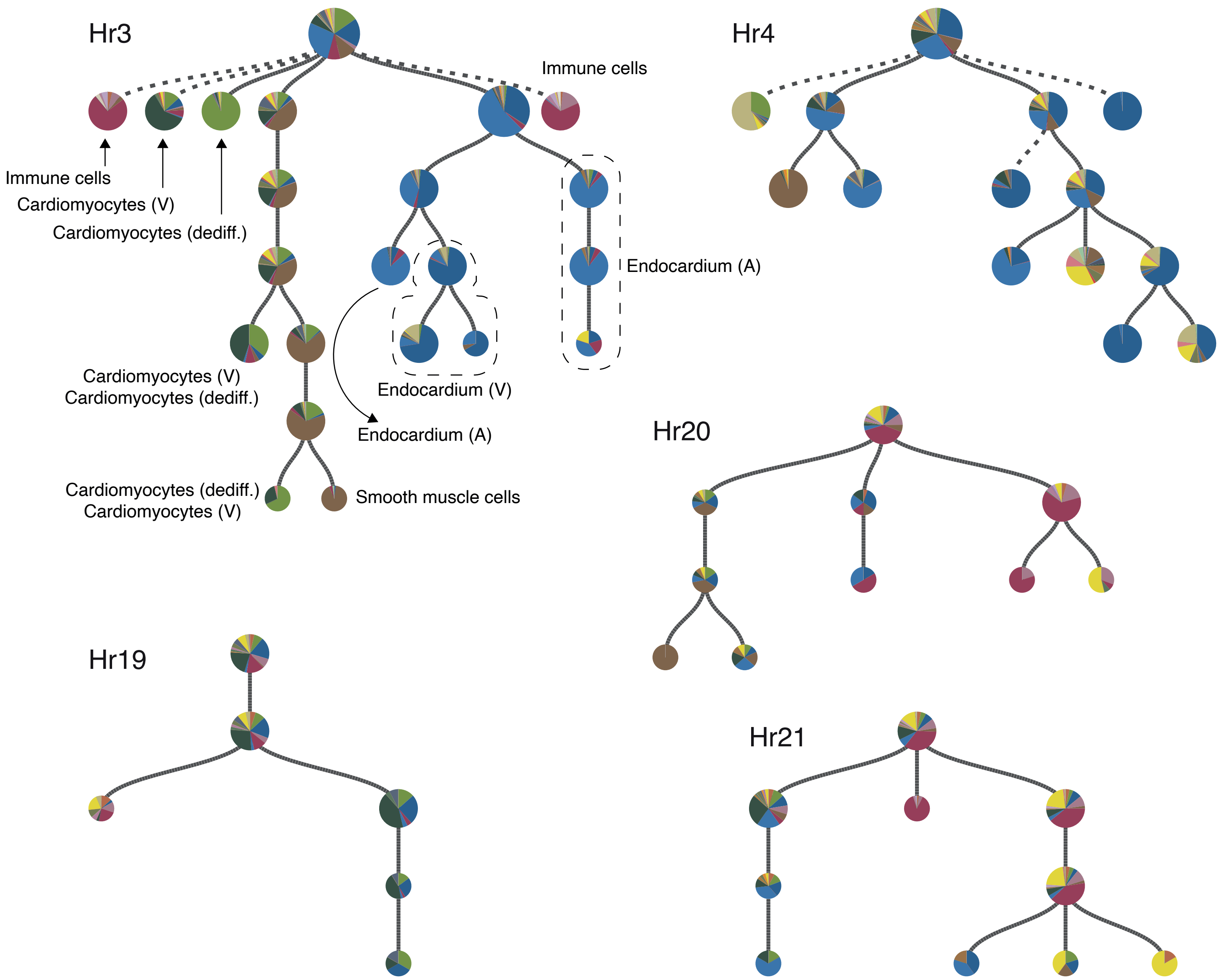

Figure S17. Lineage trees thirty days post injury. See Fig. S14 for cell type colors. In Hr3, branches are annotated by majority cell type.

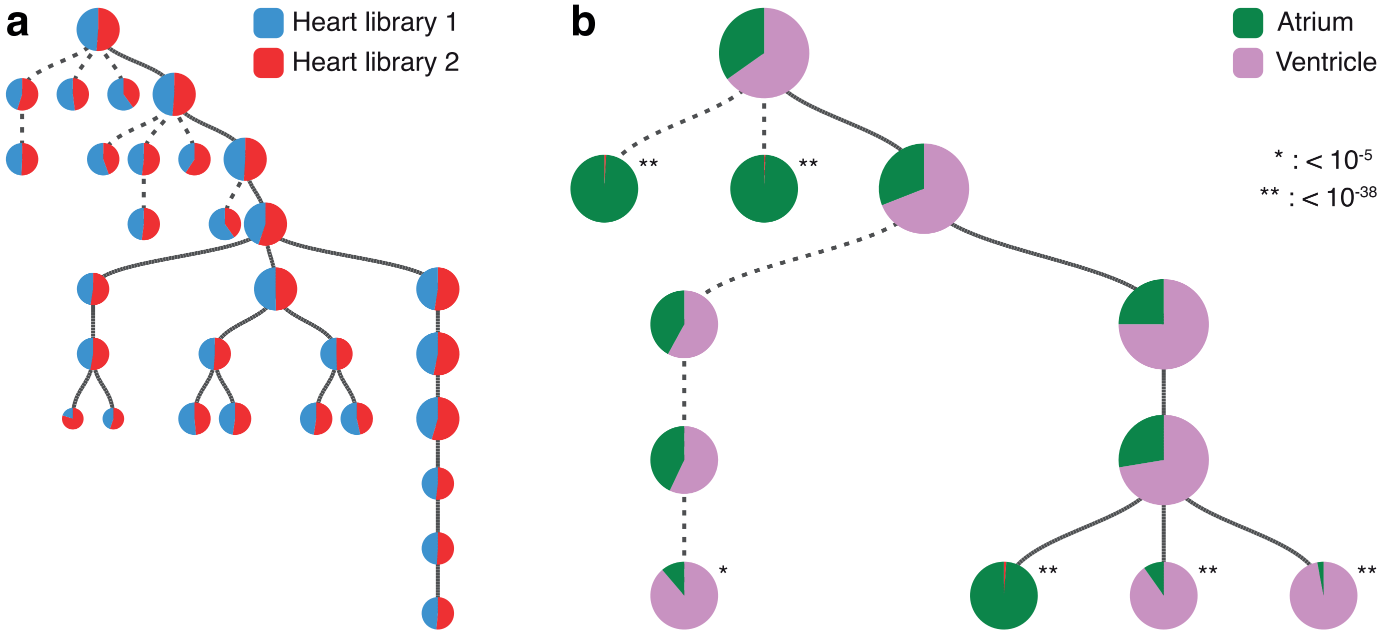

Figure S18. Technical validation of lineage trees using split libraries. (a) Randomly split libraries of a single heart (Hr2) show no (out of 14) leaf nodes significantly enriched for either library (binomial test with ratio of library sizes as null hypothesis, p-values after Benjamini-Hochberg correction for multiple testing > 0.4)**. (b)** Atrial/ventricular split libraries of a single heart (H8) show all 6 leaf nodes significantly enriched for either library (binomial test as in **a**, p-values after Benjamini-Hochberg correction for multiple testing indicated in figure).

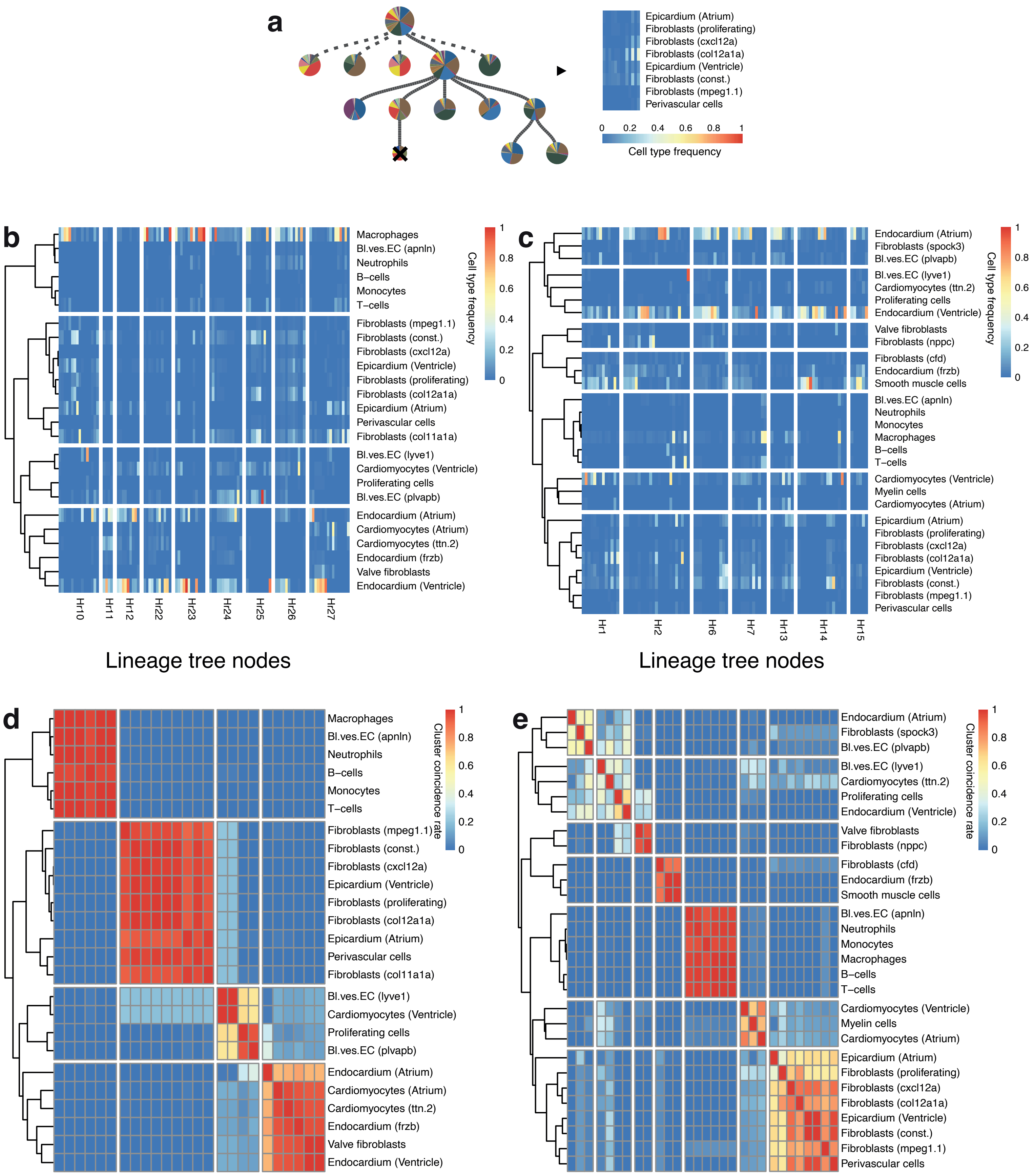

Figure S19. Identification of lineage-related cell types at 3dpi and 7dpi. (a) Vertical node removal for cell type frequency determination (see Methods). **(b-c)** Cell type frequencies per node in 3dpi **(b)** and 7dpi **(c)** trees. **(d-e)** Clustering at 3dpi **(d)** and 7dpi **(e)** is stable under 50% downsampling of cells.

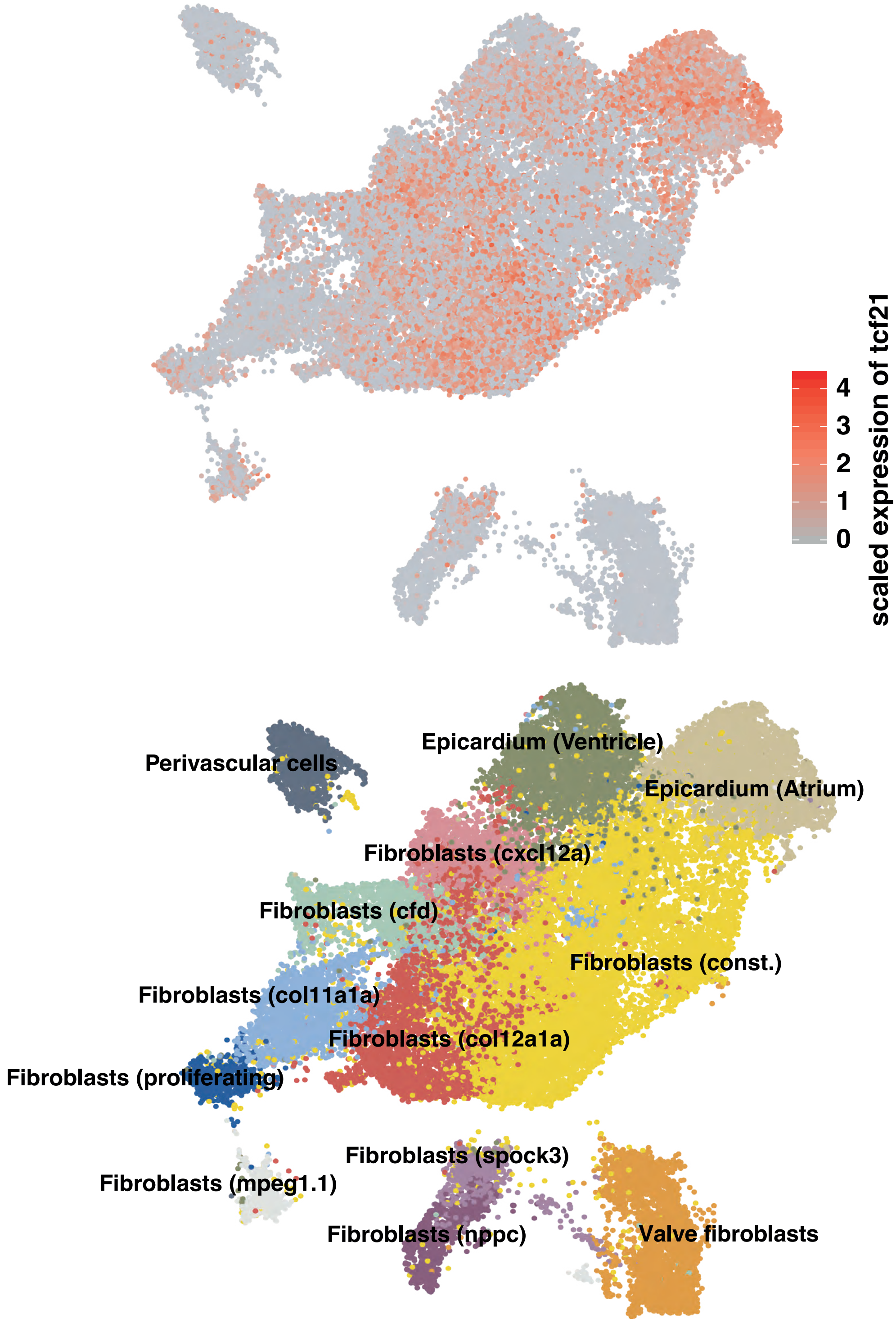

Figure S20. Expression pattern of *tcf21*. *Tcf21* is expressed in epicardial cells, constitutive fibroblasts and other fibroblast types of the epicardial cluster.

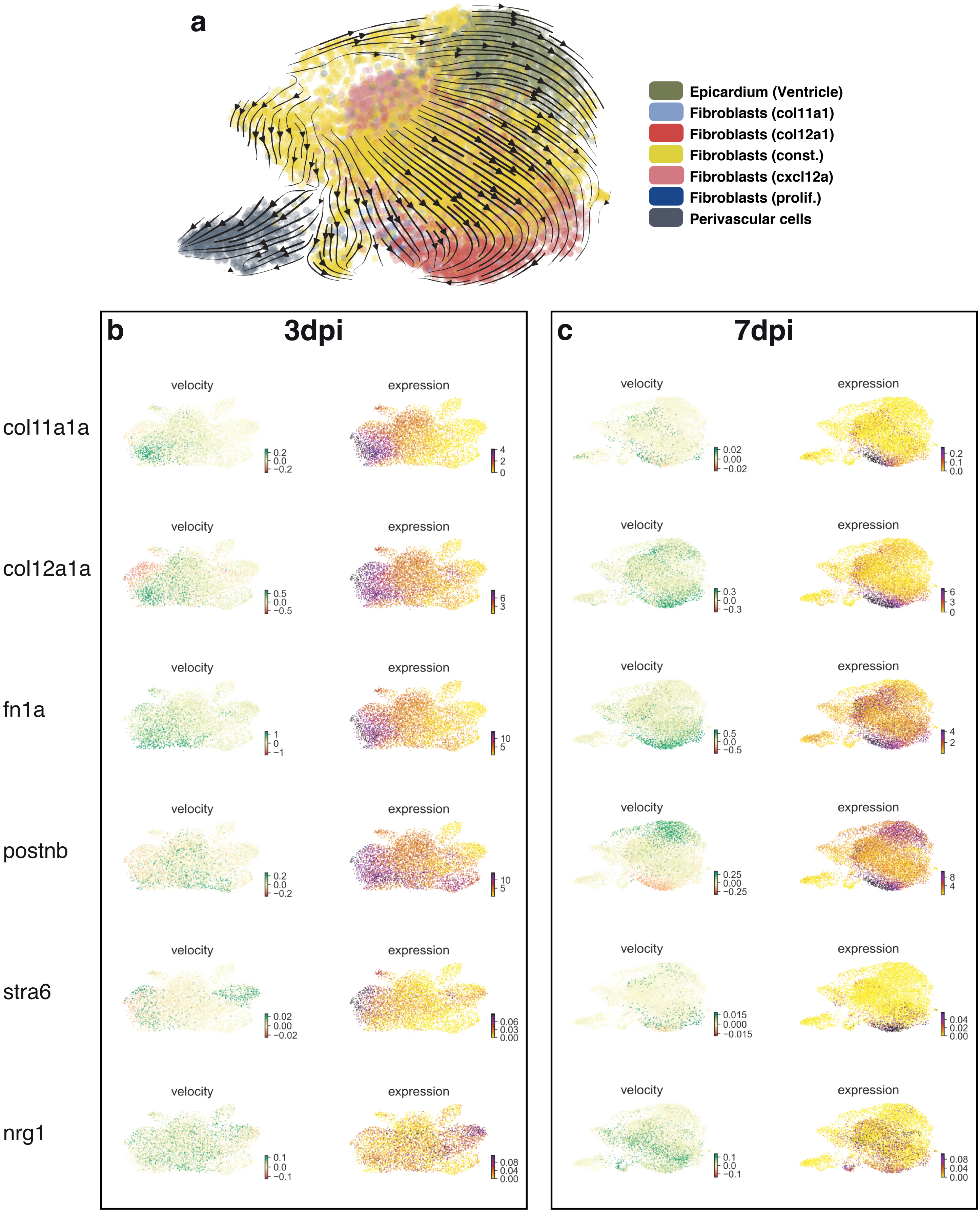

Figure S21. RNA velocity analysis of the 3 dpi and 7 dpi epicardial niche. (a) RNA velocity at 7 dpi implies constitutive fibroblasts as a source of *col12a1a* fibroblasts at 7 dpi. **(b-c)** Increase of marker genes towards niche fibroblasts at 3 dpi **(b)** and 7 dpi **(c)**. Velocity is indicated as fraction of gene transcripts generated per unit time.

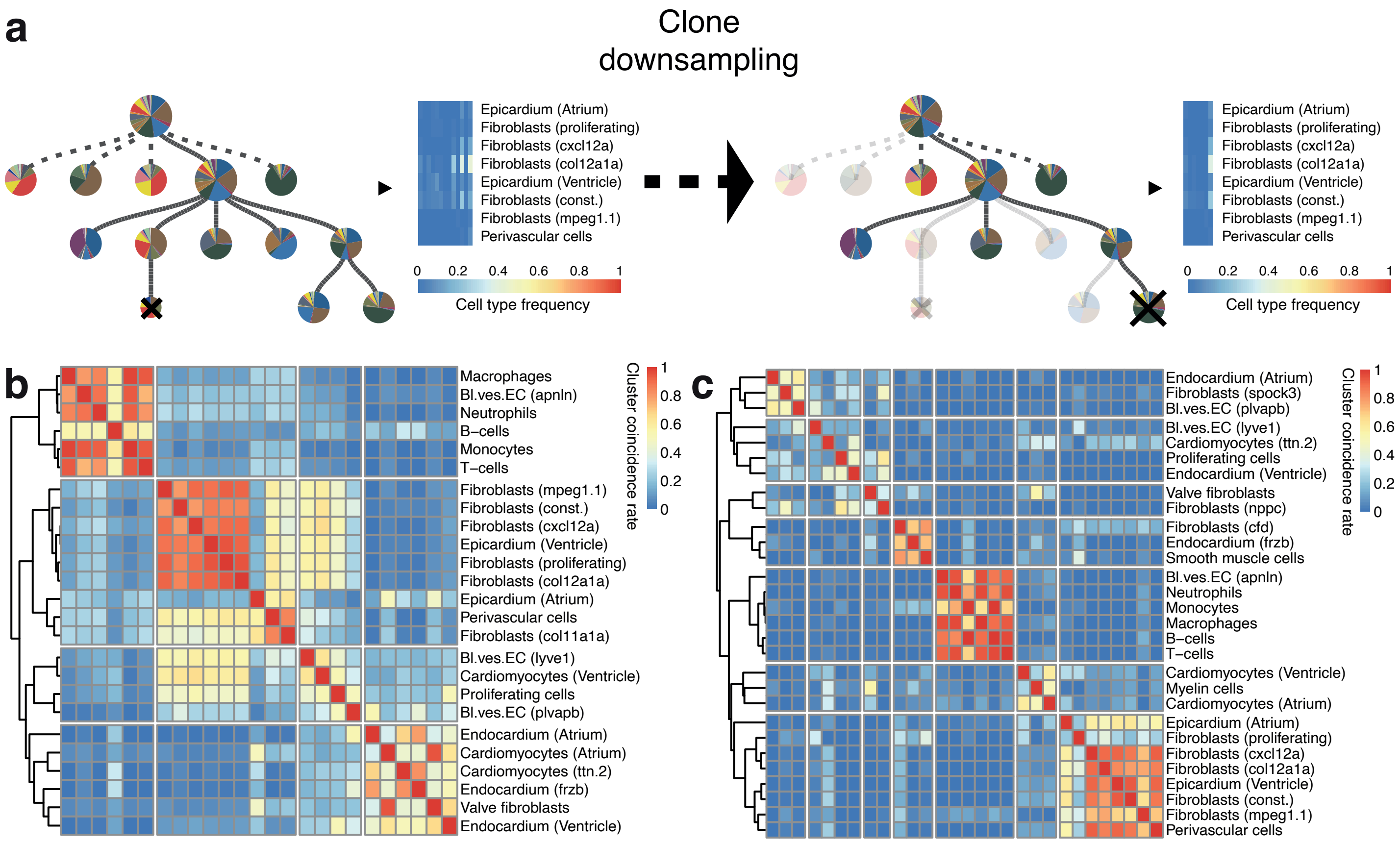

Figure S22. (a) Clone downsampling procedure. Downsampling clones shows instability of some clusters in lineage tree correlation analysis at 3 dpi **(b)** and 7 dpi **(c).**

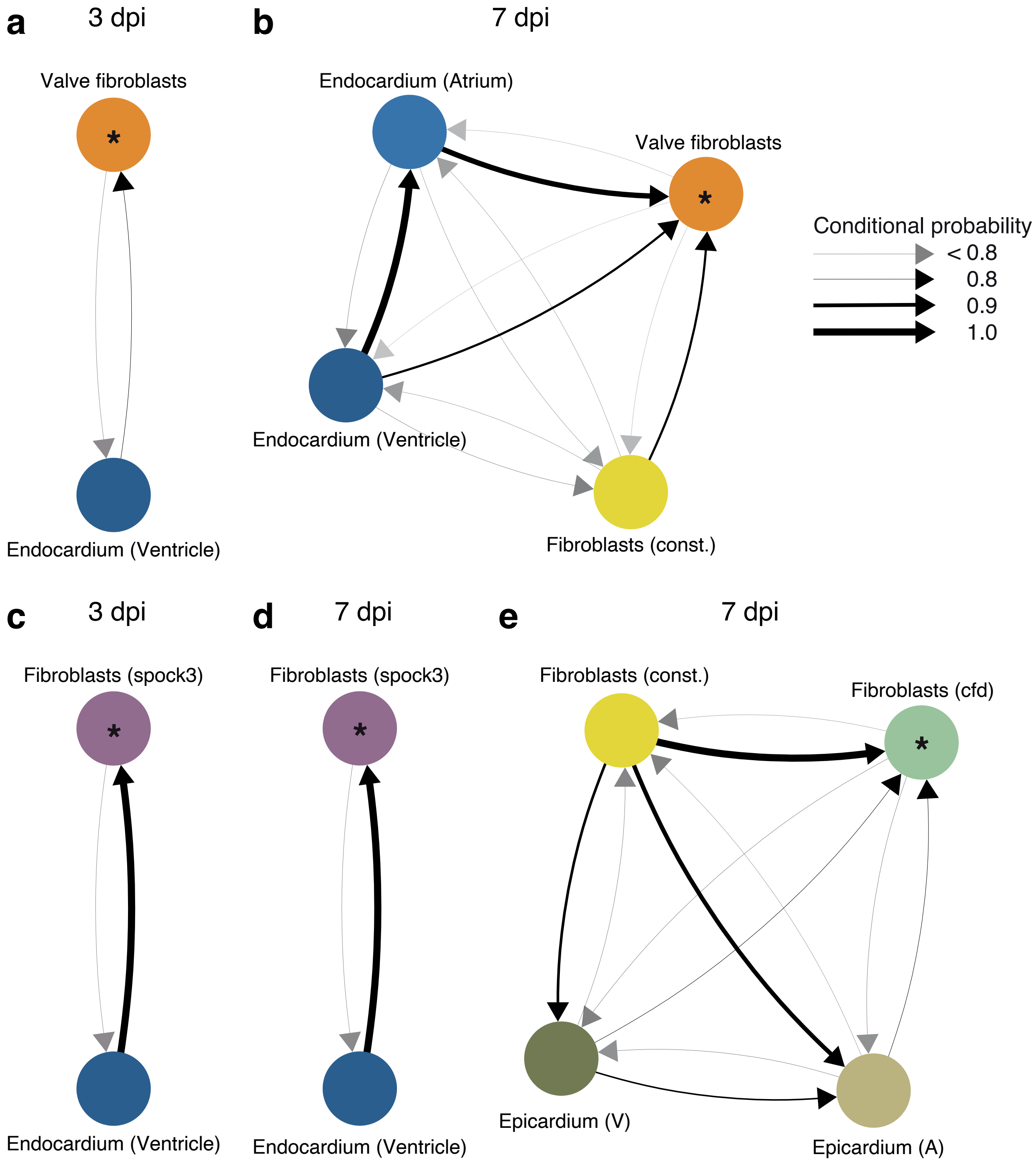

Figure S23. Lineages analyzed with conditional probability. (a) and **(b)** Valve fibroblasts share a lineage with the endocardium, observed at 3 dpi (a) and 7 dpi (b). **(c)** and **(d)** Fibroblasts (spock3) share a lineage with the endocardium, observed at 3 dpi (c) and 7 dpi (d). **(e)** Fibroblasts (cfd) share a lineage with the epicardium, shown at 7 dpi. Asterisks denote queried cell type.

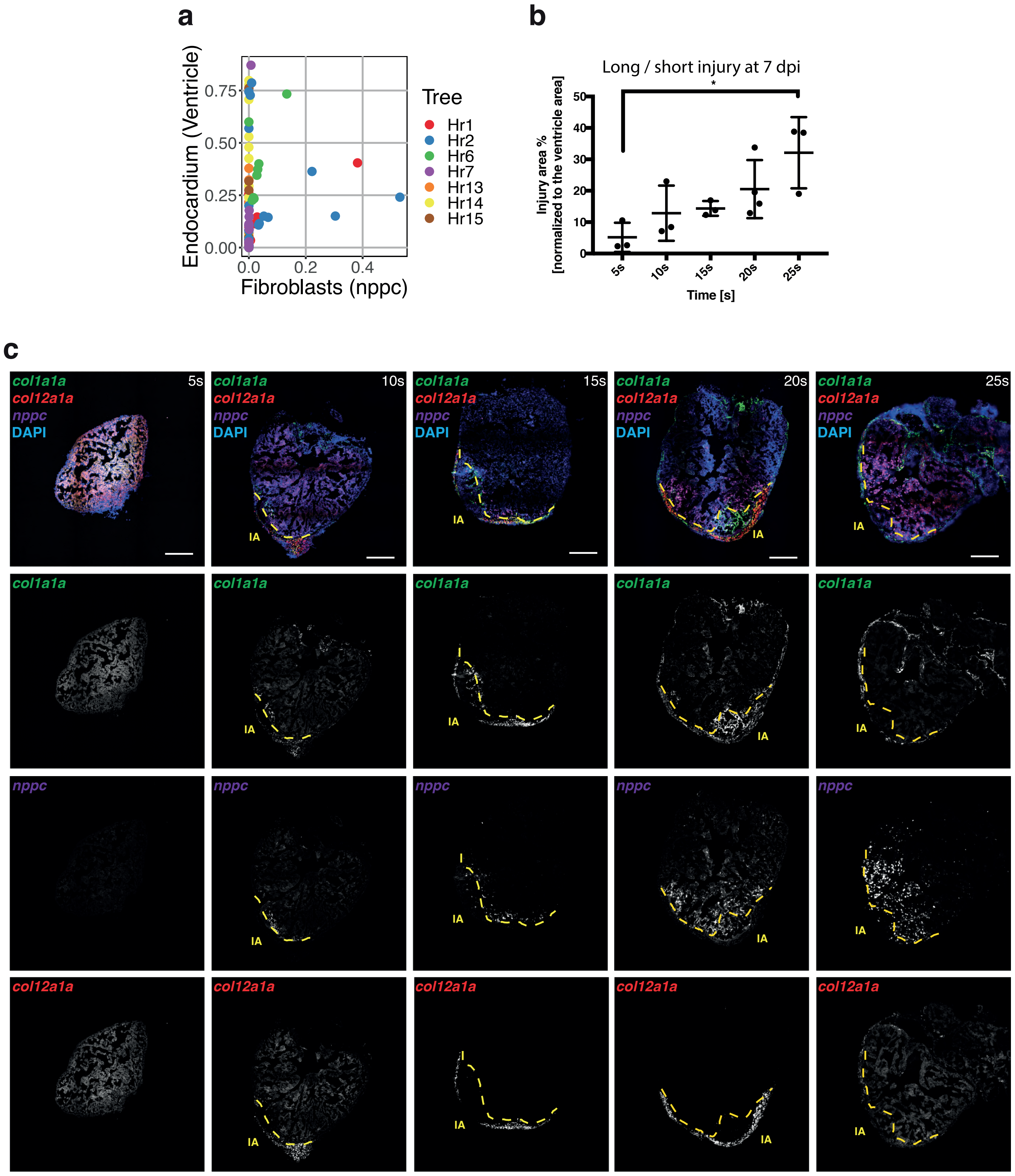

Figure S24. Longer injuries cause higher *nppc* expression. (a) Ratio between *nppc* fibroblasts and ventricular endocardium in tree nodes varies per sample. **(b)** Quantification of long and short injuries at 7dpi. Injury areas (IA) in % were normalized to the ventricular area. (n_{5s}=3, n_{10s}=3, n_{15s}=3, n_{20s}=4, n_{25s}=3). Mean ± SD. *One-way ANOVA with Tukey's multiple comparison test. *P=0.0128. **(c)** Injuries of 20s (standard procedure) and 25s penetrate deeper through heart cell layers and generate *nppc* expression in the endocardium. Scale bar: 100 μm.

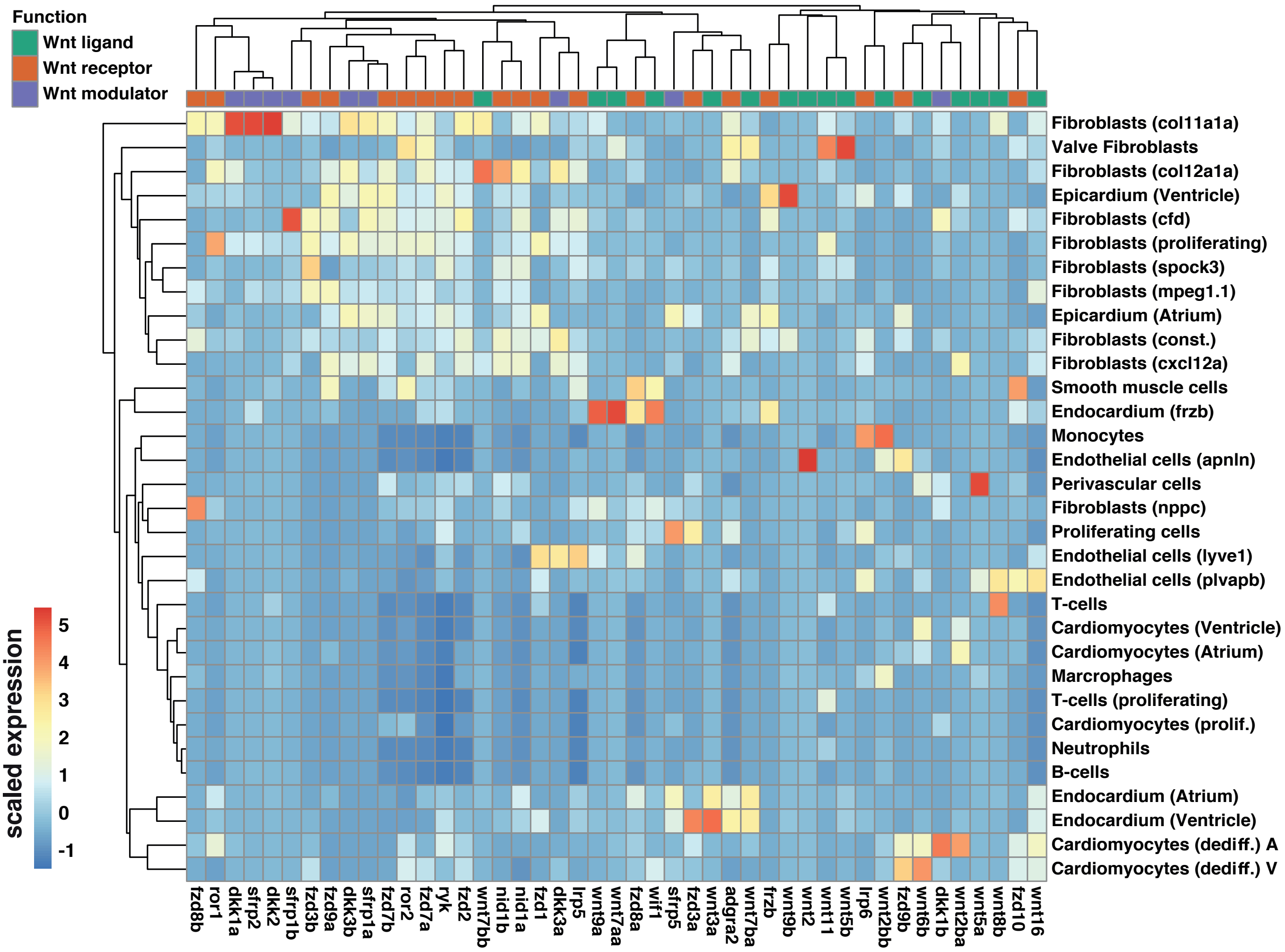

Figure S25. Expression of Wnt signaling factors in the different cell types of the zebrafish heart. Same analysis as in Fig. 6a, but with sub-cell type resolution.

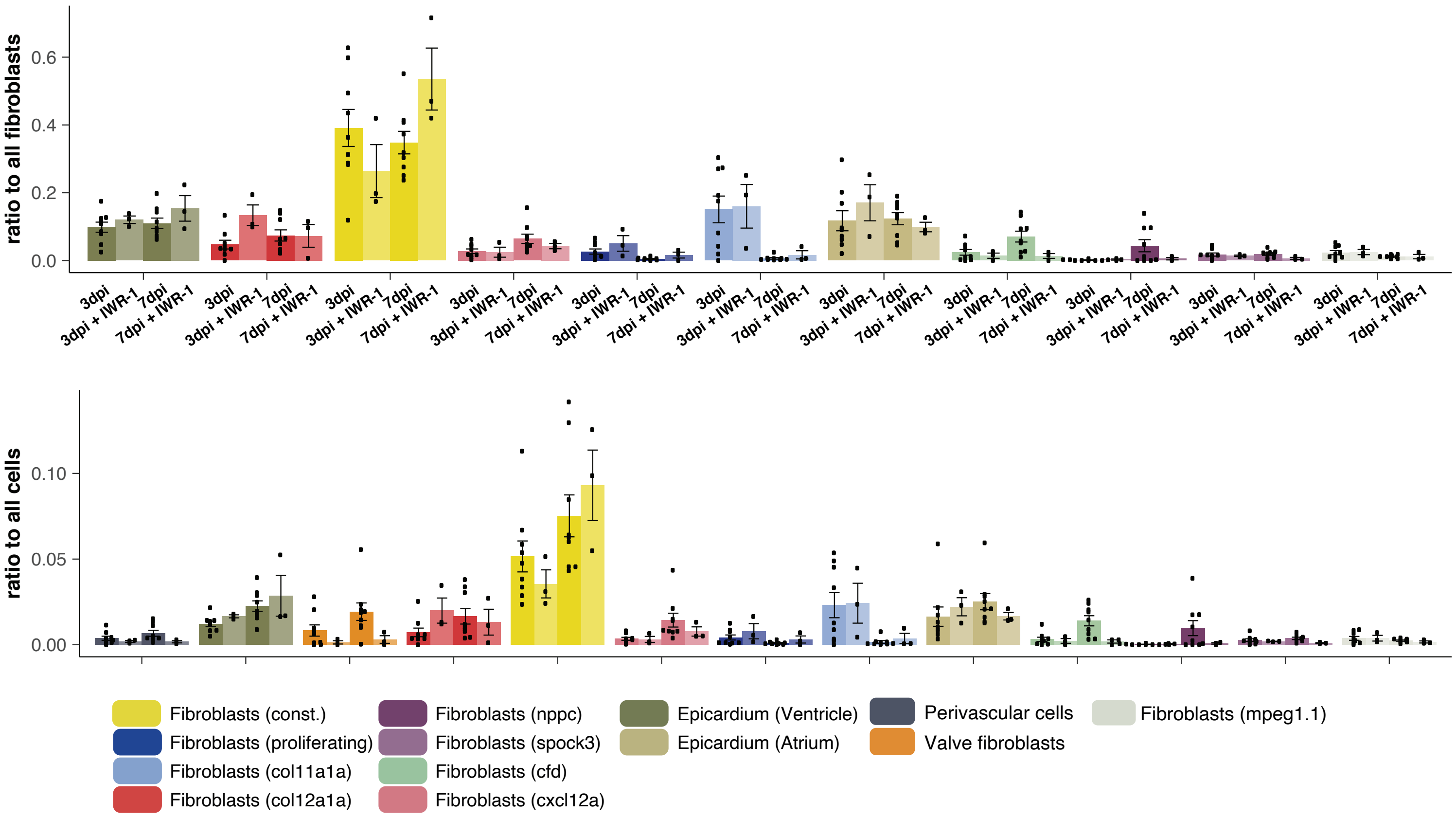

Figure S26. Comparison of cell type abundance for all fibroblast subtypes at 3 and 7 dpi with or without Wnt inhibition. Two types of normalization are shown: either cell numbers were normalized to total number of fibroblasts, or to total number of cells. Mean value across all replicates of the same treatment and timepoint is shown, error bars indicate standard error of the mean (n = 9, 3, 9, 3 animals). The three transient endocardial fibroblast types (*nppc*, *spock3*, valve fibroblasts) are strongly reduced upon Wnt inhibition, while the abundance of most other cell types does not change drastically. Of note, the *col12a1a* fibroblasts are increased at 3 dpi but reduced at 7 dpi (when normalized to all cells), indicating that *col12a1a* fibroblast abundance peaks earlier upon Wnt inhibition. *Cfd* and *cxcl12a* fibroblast abundance is transiently reduced in untreated hearts at 3 dpi (Fig. S4), and this transient depletion seems to be extended to 7 dpi upon Wnt inhibition.

Supplementary table 1: Probes for RNAscope assay

Gene Name	Accession Number	Company	Catalog #
col1a1a	NM_199214.1	ACD	409491
col11a1a	XM_005162814.1	ACD	803311-C3
col12a1a	XM_002665259.6	ACD	556481-C2
nppc	NM_001109940.1	ACD	556501-C3
cxcl12a	NM_178307.2	ACD	406481-C2
s100a10a	NM_001005961.2	ACD	556491-C2
itm2cb	NM_199980.1	ACD	556521-C3
ttn.2	XM_021479070	ACD	810421
pdgfrb	NM_001190933.1	ACD	493921-C2
angptl7	NM_001006073	ACD	845191-C3
cyp26b1	NM_212666.1	ACD	571281-C2
aldh1a2	NM_131850.1	ACD	455681-C3
pcna	NM_131404.2	ACD	574931-C3
mCherry	N/A	ACD	513201
mRFP	N/A	ACD	471271
nppa	NM_198800.3	ACD	1105441-C2

Statistical source data for Supplementary Figures S1-S26

Figure	Data sources	Notes
S1	H5, H6, H7, H8A, H8V,	All single-cell mRNA datasets
S2	H5, H6, H7, H8A, H8V	Single-cell mRNA datasets at control
S3	H5, H6, H7, H8A, H8V,	All single-cell mRNA datasets
S4	H5, H6, H7, H8A, H8V,	All single-cell mRNA datasets
S5	H5, H6, H7, H8A, H8V,	All single-cell mRNA datasets
S6	H5, H6, H7, H8A, H8V,	All single-cell mRNA datasets
S9	H5, H6, H7, H8A, H8V,	All single-cell mRNA datasets
S10	H5, H6, H7, H8A, H8V,	Single-cell mRNA datasets at control, 3 dpi and 7 dpi
S11	H5, H6, H7, H8A, H8V,	Single-cell mRNA datasets at control, 3 dpi and 7 dpi
S13	Hr1, Hr2a, Hr2b, Hr6A,	Single-cell mRNA datasets at 3 dpi and 7 dpi
S14	H5, H6, H7, H8A, H8V,	Single-cell control datasets with mRNA and lineage (scar) readout
S15	Hr10, Hr12, Hr22, Hr24	Single-cell 3 dpi datasets with mRNA and lineage (scar) readout
S16	Hr1, Hr2a, Hr2b, Hr6A,	Single-cell 7 dpi datasets with mRNA and lineage (scar) readout
S17	Hr3, Hr4, Hr19, Hr20, H	Single-cell 30 dpi datasets with mRNA and lineage (scar) readout
S18A	Hr2a, Hr2b, Hr2 scar	Dataset of a randomly split single-cell suspension with mRNA and lineage readout
S18B	H8A, H8V, H8 scar	Dataset of an atrial/ventricular split single-cell suspension with mRNA and lineage readout
S19B, D	Hr1, Hr2a, Hr2b, Hr6A,	Single-cell 7 dpi datasets with mRNA and lineage (scar) readout
S19A	Hr1, Hr1 scar	Single-cell dataset with mRNA and lineage readout
S19C, E	Hr10, Hr12, Hr22, Hr24	Single-cell 3 dpi datasets with mRNA and lineage (scar) readout
S20	H5, H6, H7, H8A, H8V,	All single-cell mRNA datasets
S21A, C	Hr1, Hr2a, Hr2b, Hr6V,	Single-cell uninhibited mRNA datasets at 7 dpi with >50 fibroblasts (nppc)
S21B	Hr10, Hr12, Hr22, Hr24	All uninhibited single-cell 3 dpi mRNA datasets
S22A	Hr1, Hr1 scar	Single-cell dataset with mRNA and lineage readout
S22B	Hr10, Hr12, Hr22, Hr24	Single-cell 3 dpi datasets with mRNA and lineage (scar) readout
S22C	Hr1, Hr2a, Hr2b, Hr6A,	Single-cell 7 dpi datasets with mRNA and lineage (scar) readout
S23A, C	Hr10, Hr12, Hr22, Hr24	Single-cell 3 dpi datasets with mRNA and lineage (scar) readout
S23B, D, E	Hr1, Hr2a, Hr2b, Hr6A,	Single-cell 7 dpi datasets with mRNA and lineage (scar) readout
S24A	Hr1, Hr2a, Hr2b, Hr6A,	Single-cell 7 dpi datasets with mRNA and lineage (scar) readout
S25	H5, H6, H7, H8A, H8V,	All uninhibited single-cell mRNA datasets
S26	Hr1, Hr2a, Hr2b, Hr6A,	Single-cell mRNA datasets at 3 dpi and 7 dpi

S12F	Normalized injury area		*Tg(-4kbcol12a1:GAL4VP16;UAS:NTR:RFP)*
	Experiment 1	DMSO	MTZ-treated 7 dpi
		30.40	32.25
		22.90	43.30
			22.97
	Experiment 2	29.60	20.39
		21.46	25.23
			30.28

S24B	Normalized injury area		AB				
	Experiment 1	5s	10s	15s	20s	25s	7 dpi
		10.54	22.99	13.81	19.51	38.49	
		2.35	8.45	16.95	12.89	38.81	
		2.65	7.14	12.33	15.90	19.03	
					33.78		
