# Supplementary material for: Origin and function of activated fibroblast states during zebrafish heart regeneration
Source: Nat Genet. 2022 Jul 21;54(8):1227–37. doi: 10.1038/s41588-022-01129-5 (PMC7613248; doi:10.1038/s41588-022-01129-5)
Supplement: Supplementary file 2 — Reporting Summary [file 41588_2022_1129_MOESM2_ESM.pdf]

nature research

Corresponding author(s): Jan Philipp Junker, Daniela Panakova

Last updated by author(s): May 5, 2022

Reporting Summary

Nature Research wishes to improve the reproducibility of the work that we publish. This form provides structure for consistency and transparency in reporting. For further information on Nature Research policies, see our Editorial Policies and the Editorial Policy Checklist.

Statistics

For all statistical analyses, confirm that the following items are present in the figure legend, table legend, main text, or Methods section.

n/a	Confirmed	
☐	☒	The exact sample size (*n*) for each experimental group/condition, given as a discrete number and unit of measurement
☒	☐	A statement on whether measurements were taken from distinct samples or whether the same sample was measured repeatedly
☐	☒	The statistical test(s) used AND whether they are one- or two-sided<br>*Only common tests should be described solely by name; describe more complex techniques in the Methods section.*
☒	☐	A description of all covariates tested
☐	☒	A description of any assumptions or corrections, such as tests of normality and adjustment for multiple comparisons
☐	☒	A full description of the statistical parameters including central tendency (e.g. means) or other basic estimates (e.g. regression coefficient) AND variation (e.g. standard deviation) or associated estimates of uncertainty (e.g. confidence intervals)
☐	☒	For null hypothesis testing, the test statistic (e.g. *F*, *t*, *r*) with confidence intervals, effect sizes, degrees of freedom and *P* value noted<br>*Give P values as exact values whenever suitable.*
☒	☐	For Bayesian analysis, information on the choice of priors and Markov chain Monte Carlo settings
☐	☒	For hierarchical and complex designs, identification of the appropriate level for tests and full reporting of outcomes
☐	☒	Estimates of effect sizes (e.g. Cohen's *d*, Pearson's *r*), indicating how they were calculated

Our web collection on statistics for biologists contains articles on many of the points above.

Software and code

Policy information about availability of computer code

Data collection	Zen 2.3 SP1 FP3 (version 14.0.0.0) and Zen 3.2 blue edition were used to acquire confocal images.<br>BIOREVO Keyence BZ-9000 Viewer was used to acquire histological images.
Data analysis	Cell Ranger (version 3.0.2) was used to align and demultiplex single-cell transcriptomics data.<br>bwa mem (version 0.7.12) was used to map scar data.<br>Velocyto (version 0.17) was used to extract mRNA velocity data from single-cell transcriptomics data.<br>All data analysis was done in R (version 3.3 and higher), except when indicated.<br>The R package Seurat (version 3.0) was used to cluster single-cell transcriptomics data.<br>Interactions between cell types were calculated using CellPhoneDB (https://github.com/Teichlab/cellphonedb) (version 2.1.4) and further analyzed in custom scripts provided at https://github.com/Bastiaanspanjaard/Heart_regeneration.<br>Analysis of cellular trajectories and mRNA velocity were done in Python (3.8) using scanpy (version 1.8.2) and scvelo (version 0.2.4).<br>Deconvolution of TOMO-seq data was done in Python (3.8) using AutoGeneS (https://github.com/theislab/AutoGeneS) (version 1.0).<br>We developed custom code to filter, analyze and visualize scar data. The custom code is provided at https://github.com/Bastiaanspanjaard/Heart_regeneration.<br>Microscopy data was analyzed in Fiji/ImageJ including the Particle analyzer plug-in (version 2.1.0) and Adobe Photoshop (version 20.0.6).<br>GraphPad Prism 7/9 (version 7.0d/9.3.1) was used for plotting graphs and statistical analysis.

For manuscripts utilizing custom algorithms or software that are central to the research but not yet described in published literature, software must be made available to editors and reviewers. We strongly encourage code deposition in a community repository (e.g. GitHub). See the Nature Research guidelines for submitting code & software for further information.

Data

Policy information about availability of data

All manuscripts must include a data availability statement. This statement should provide the following information, where applicable:

- Accession codes, unique identifiers, or web links for publicly available datasets
- A list of figures that have associated raw data
- A description of any restrictions on data availability

Sequencing data are deposited on Gene Expression Omnibus, accession numbers GSE159032 and GSE158919. Transcriptome data was aligned to a zebrafish transcriptome created with Cell Ranger 3.0.2 from GRCz11, release 92. We performed an orthologue conversion of the Vertebrate Secretome Database VerSeDa, using orthology data from the Alliance of Genome Resources, release 3.2.0.

Field-specific reporting

Please select the one below that is the best fit for your research. If you are not sure, read the appropriate sections before making your selection.

☒ Life sciences ☐ Behavioural & social sciences ☐ Ecological, evolutionary & environmental sciences

For a reference copy of the document with all sections, see nature.com/documents/nr-reporting-summary-flat.pdf

Life sciences study design

All studies must disclose on these points even when the disclosure is negative.

Sample size	The number of cells to be analyzed was based on the maximum number attainable. Since the various detected cell types have vastly different abundances, we focused the analysis on those cell types that were detected at high enough numbers for statistical analysis.<br>Sample size for animal test groups was determined using G*Power calculator (G*Power 3.1).
Data exclusions	No data was excluded. However, the following filtering steps were applied to the scar sequencing data in order to mitigate the effect of sequencing errors, PCR amplification errors and formation of chimeric reads: removal of all sequences that were detected only once; removal of molecules with the same BC+UMI but different scar, same BC+scar but different UMI, and same UMI+scar but different BC; removal of scars with a Hamming distance ≤2 to a more highly detected scar; removal of the most lowly detected scars; and removal of cells that express more scars than expected (putative doublets).<br><br>For lineage analysis, only scars with creation probability <0.01 (as determined by bulk experiments) were used in order to minimize artifacts caused by repeated creation of the same scar sequence in one animal.
Replication	All attempts at replication were successful. The number of replicate experiments is provided in figure legends and/or the section "Statistics and reproducibility" in the Methods
Randomization	Formal randomization of samples was not applicable, since no manual selection of single cells was performed during experiments and analysis.<br>Animals were randomly assigned to test groups.
Blinding	Formal blinding was not applicable, since no manual selection of single cells was performed during experiments and analysis. Data collection and analysis were not performed blind to the conditions of the experiments. Sample collection and genomics analysis were performed by two independent experimenters.

Reporting for specific materials, systems and methods

We require information from authors about some types of materials, experimental systems and methods used in many studies. Here, indicate whether each material, system or method listed is relevant to your study. If you are not sure if a list item applies to your research, read the appropriate section before selecting a response.

Materials & experimental systems

n/a	Involved in the study	
☒	☐	Antibodies
☒	☐	Eukaryotic cell lines
☒	☐	Palaeontology and archaeology
☐	☒	Animals and other organisms
☒	☐	Human research participants
☒	☐	Clinical data
☒	☐	Dual use research of concern

Methods

n/a	Involved in the study	
☒	☐	ChIP-seq
☒	☐	Flow cytometry
☒	☐	MRI-based neuroimaging

Animals and other organisms

Policy information about studies involving animals; ARRIVE guidelines recommended for reporting animal research

Laboratory animals	Zebrafish (Danio rerio, strain AB). Adult zebrafish of random sex, aged between 4 months and a year, and with a length of at least 3 cm were used.
Wild animals	No wild animals were used in this study.
Field-collected samples	No field collected samples were used in this study.
Ethics oversight	Zebrafish were bred, raised and maintained in accordance with the FELASA guidelines, the guidelines of the Max-Delbrück Center for Molecular Medicine and the Max Planck Society, and the local authorities for animal protection (Landesamt für Gesundheit und Soziales, Berlin, and The Veterinary department of the Regional Board of Darmstadt, Darmstadt, Germany) for the use of laboratory animals based on the current version of German law on the protection of Animals and EU directive 2010/63/EU on the protection of animals used for scientific purposes. In addition, housing and breeding standards followed also the international 'Principles of Laboratory Animal Care' (NIH publication no. 86-23, revised 1985).

Note that full information on the approval of the study protocol must also be provided in the manuscript.
